# LungCraft: a hybrid 3D-2D deep learning and radiomics framework with explainable AI for precision diagnosis of lung cancer

**DOI:** 10.3389/frai.2026.1853361

**Published:** 2026-06-23

**Authors:** Nithin Kodipyaka, G. Suganya, Deepika Roselind Johnson

**Affiliations:** School of Computer Science and Engineering, Vellore Institute of Technology, Chennai, India

**Keywords:** 3D modelling, CT visualization, explainable AI, grad-CAM, HybridNET, lung adenocarcinoma, lung-cancer diagnosis, quantitative imaging biomarkers

## Abstract

**Background and objective:**

Lung cancer is the top cause of cancer-related death, globally. The morphological complexity of tumours, intra-tumoural heterogeneity and limited interpretability of current imaging systems all contribute to the difficulty of early and reliable detection of tumours. This paper introduces LungCraft, a diagnostic framework composed of 3D medical modelling, quantitative radiomics and explainable artificial intelligence (XAI) for lung adenocarcinoma classification, using computed tomography (CT) scans as input.

**Methods:**

LungCraft uses a hybrid deep learning architecture (HybridNET) which has a combination of layers that use 3D convolutional layers for volumetric feature learning and 2D convolutional layers for contextual refinement. Sixty-one TCIA CT volumes were used for internal validation, while the LIDC-IDRI and NSCLC-Radiomics datasets were kept as held-out test sets for external validation. The spatial and feature level interpretability is given by Grad-CAM and KernelSHAP. Unlike previous methods which focussed on achieving one or more of these, LungCraft’s main strength is that it brings these in a systematic way as part of an integrated pipeline that includes a two-stage feature-level fusion strategy, dual spatial and feature level explainability and interactive 3D tumour visualization.

**Results:**

On internal test set, LungCraft was able to classify with an accuracy of 91.3% and an AUC of 0.93, whereas on external cohorts it was able to classify with an accuracy of 88.7% and an AUC of 0.91 (LIDC-IDRI) and 89.6% and 0.92 (NSCLC-Radiomics). 95% confidence intervals are given for all measurements. The results are statistically significant (*p* < 0.01) when compared with the results of the baseline architecture.

**Conclusion:**

LungCraft achieves accurate and interpretable diagnosis of lung adenocarcinoma, with demonstrated external generalisation and has a potential use as an AI assistant in clinical decision making which is subject to formal prospective validation.

## 1 Introduction

Lung adenocarcinoma is among the most clinically significant malignancies globally, accounting for approximately 18% of all cancer-related deaths. Computed tomography (CT) is the most used clinical imaging setting as it is based on evolution of many imaging modalities, which are two-dimensional (2D). Volumetric CT is a popular imaging modality in clinical practice that offers detailed three-dimensional spatial data of lung structures. Using 2D cross-sections, radiologists are able to determine benign and malignant tumours with high confidence and even determine the growth of tumours over time with better precision.

Radiological assessment is commonly performed through 2D slice-wise interpretation, which can limit appreciation of complex tumour morphology and spatial heterogeneity. It can restrict the overall knowledge of the intricate tumour morphology and spatial heterogeneity. Although, current deep learning models diagnose and identify lung cancer, they have not been fully applied in clinical practice due to interpretability, transparency and generalizability issues. The lack of explainability diminishes clinician confidence and transparency, it enforces the need for clinically aligned AI models. Apart from improving diagnostic accuracy, imaging biobanks provide structured repositories which integrate medical images with imaging biomarkers and patient clinical data. This can be used as the foundation of large-scale analysis and precision medicine. Although it is increasingly important, a small share of current biobanks include imaging data, which underlines the necessity of AI-based frameworks to promote precision medicine (Gabelloni et al., 2022).

To address these limitations, this paper proposes LungCraft, a hybrid 3D-XAI diagnostic framework. It improves diagnostic performance and understanding via interactive 3D visualization, hybrid convolutional-based modelling and explainable artificial intelligence. Firstly, the Marching Cubes algorithm enables three-dimensional volumetric reconstruction of a specified CT scan, which enables physicians to view the tumour in any angle with per density gradient and volumetric irregularities. Secondly, a set of imaging biomarkers are quantitatively obtained to examine the tumour shape and intratumour heterogeneity that have been statistically linked to the survival of lung adenocarcinoma in the past.

Although individual components of LungCraft (3D CNNs, radiomics, Grad-CAM and SHAP) have been individually reported in previous literature, the novelty of this study is the systematic and purposeful combination of these components into a single unified diagnostic pipeline, as well as the specific design choices that set LungCraft apart from previous works. Below we specify five exact contributions, which we elaborate and separate from the closest related work.

- First, the use of a hybrid architecture with (3D) convolutional stream for volumetric spatial learning and (2D) EfficientNet-B0 stream for contextual slice-level feature extraction for lung adenocarcinoma classification has not been previously reported. Existing hybrid methods like Li et al. (2024) that use handcrafted and deep features are all 2D-based and 3D CNN methods like Alakwaa et al. (2017) and Wu et al. (2023) do not apply 2D contextual refinement. On one hand, the specific design of HybridNET, the concatenation of the two stream outputs followed by an additional normalized radiomic vector enables the simultaneous encoding of volumetric geometry, contextual texture and quantitative morphology, none of which are encoded by any previous single framework in this space.
- Secondly, the use of quantitative radiomics at the feature level as opposed to being a standalone classifier distinguishes LungCraft from radiomics-only pipelines like Zhang et al. (2023) and Adiraju et al. (2025), which do not use deep learning methods and from deep learning pipelines like Hammad et al. (2025) and Kumaran et al. (2024), which do not feature handcrafted radiomic descriptors. Unlike the decision-level ensemble fusion, feature-level fusion also allows the fully connected classification head to learn the cross-modal interaction between deep spatial embedding and radiomic descriptors.
- Third, the dual explainability of the Grad-CAM spatial localization and KernelSHAP feature attribution presents both a ‘where’ and a ‘why’ explainability within one model. Previous XAI strategies for lung cancer diagnosis use only one of either spatial or feature attribution approaches separately, but not both. The novelty of the method lies in the fact that Grad-CAM operates on the convolutional streams to localize diagnostically relevant spatial regions, that KernelSHAP operates on the radiomic part of the fused representation to quantify the contribution of handcrafted descriptors and that these two explanation types are complementary and not redundant.
- Fourth, an interactive 3D visualization module (Marching Cubes algorithm) lets the clinician rotate and slice the tumour, while also showing the Grad-CAM activations on the 3D Marching Cubes mesh instead of on the 2D axial slices. This is the first lung cancer AI framework in the literature to incorporate interactive 3D tumour visualization with AI explanation overlays in a clinician-facing interface, thus directly solving the clinical adoption problem of static 2D explanation outputs that fail to capture the three-dimensional nature of the lung cancer diagnostic task.
- Fifth, the evaluation design (using a strictly held-out primary test partition with bootstrapped 95% confidence intervals, disaggregated external validation on two independent cohorts of 1,440 patients, five-fold cross-validation and a multi-reader inter-observer agreement assessment of explainability outputs) is more rigorous than that of most comparable frameworks in the literature, where external validation, if present, is either aggregated or not performed. This is a form of evaluation rigor that in and of itself is a contribution to the practice of reproducible benchmarking for AI-assisted lung cancer diagnosis.

The proposed work enhances the use of the static diagnostic imaging method to the interactive method using the assistance of explainable 3D analytics to allow physicians to gain a comprehensive understanding of both the tumour behavior and the diagnostic reasoning of the AI.

## 2 Related work

Early studies on computer-aided diagnosis (CAD) were focused on automated analysis of lung imaging, specifically on the issue of segmentation, feature extraction and early disease detection in thoracic imaging.

### 2.1 Computer-aided diagnosis and 3D lung modelling

El-Baz et al. (2013) created a set of CAD systems to detect lung cancer through a combination of handcrafted features and segmentation-based models. These frameworks pointed to the difficulties in determining boundary detection and model generalization. This paper has highlighted the importance of reproducible and quantitative imaging biomarkers as opposed to relying on visual interpretations only. A hybrid 3D lung tissue culture extraction technique was suggested by Bailey et al. (2018) based on ex vivo models of chronic obstructive pulmonary disease. This method showed the possibility of combining biological and computational 3D modelling to recreate the real lung conditions. Ikeda et al. (2013) suggested an 3D CT modelling method to improve the pre-operative navigation of thoracoscopic surgery. The method improves volumetric data, anatomical understanding and increased surgical precision. Chen et al. (2017) suggested a stem-cell-based 3D lung model system to model developmental and pathological processes that effectively calculated the spatial cell-matrix interactions that subsequently improved the effect diagnostic imaging characteristics. Although, these studies emphasize the significance of 3D modelling in anatomical knowledge, they mostly do not consider data-driven deep learning and explainability processes necessary in contemporary clinical decision support.

### 2.2 Deep learning for lung cancer detection

Convolutional Neural Networks (CNNs) are deep learning methods that have improved the detection of advanced lung cancer. Alakwaa et al. (2017) proposed a new 3D-Convolutional Neural Network (3D-CNN) model of automated lung-cancer classification using CT scans that demonstrated that the 3D method is superior to the 2D method. Eldho and Nithyanandh (2024) enhanced the detection accuracy by expanding the 3D CNN architecture by applying DICOM datasets to evaluate tumour detection and its severity. Saravanaprasad and Karuppusamy (2024) introduced a 3D CAD system, which integrates segmentation and classification strategies to improve the accuracy of the diagnosis in the case of early tumour detection. Wu et al. (2023) suggested a multi-kernel 3D CNN architecture to identify the nodules in the lungs of different sizes. This method is used to handle variability in lesion size and contrast that is perceived to be a challenge. Therefore, the results of these models confirm that 3D-CNNs can be used to analyze lungs. Although, these methods have been shown to perform better, their high computational complexity and lack of interpretability tend to limit their direct clinical usefulness.

### 2.3 Hybrid and optimization-based approaches

With the growth of data availability and diagnostic expectations, researchers shifted to hybrid and optimization-based solutions. As an example, Li et al. (2024) suggested a hybrid feature-machine learning model that combines both handcrafted features and deep features and optimized hyperparameter tuning to achieve more stable predictions. Shekhar and Khetavath (2024) proposed a CNN-based approach based on Garter Snake Optimization which increased segmentation and classification accuracy. Bhuiyan et al. (2024) compared different machine learning applications to early detection using ensemble and optimization strategies to maximize sensitivity to improve the detectability of small lesions. Nusantoro et al. (2024) provided an extensive survey of different deep-learning methods of lung-cancer detection. Based on their analysis, they found that hybrid CNNs and feature fusion technique are the new standards of CT analysis. These approaches serve as architectural precursors for other frameworks such as HybridNET which is used in our proposed approach. Despite the fact that hybrid and optimization-based models enhance the predictive performance, these models are mainly aimed at enhancing accuracy and have little information on the decisions made by the models.

### 2.4 Radiomics and quantitative imaging biomarkers

Radiomics has become an effective method of deriving quantitative imaging biomarkers of CT and PET scans. Some of the authors concentrated on quantitative radiomics through the extraction of morphological, textural and other features of the CT and PET images. Zhang et al. (2023) found that the 18F-FDG PET radiomics dataset, which is trained with the help of a machine learning model can predict the presence of KRAS mutation in untreated lung adenocarcinoma disease. Adiraju et al. (2025) referred to the fact that CT-based tumour heterogeneity and tumour shape. This ensured that quantitative imaging biomarkers are efficient in increasing the accuracy of the predictions. Aguinagalde et al. (2022) offered a method of automating the volume measurement of the chest X-rays. These works prove that 3D morphological descriptors are reliable diagnostic indicators which are employed in the pipeline of our proposed work. Nevertheless, radiomics-based methods do not have hierarchical feature learning features and are not often combined with deep learning within a single framework.

### 2.5 Explainable AI in medical imaging

Explainable Artificial Intelligence (XAI) is a concept that is becoming more common in recent times to explain the predictions of black-box models. Hammad et al. (2025) suggested a CNN architecture with the use of Grad-CAM methodology in detecting lung-cancer by CT-based methodology. This framework increased the prediction accuracy by visualizing class-discriminative regions to build clinician trust. Kumaran et al. (2024) used VGG16, ResNet50 and InceptionV3 models to generate a hybrid model with Grad-CAM to generate ensemble interpretability maps to enhance classification accuracy. Fontes et al. (2024) presented a systematic review of different XAI techniques in medical imaging using both visual and case-based explanations to increase clinical credibility. Vanitha et al. (2024) integrated deep-learning models with personalized explainability modules in the classification of lung and colon cancers. The ensemble model also increased model interpretability which facilitates multi-disease generalization. Alqhatni et al. (2025) used EfficientNet integrated with Grad-CAM approach for tumour identification. Shin et al. (2024) suggested an XAI methodology to detect circulating tumour DNA detection using multimodal data fusion to improve AI interpretability. Such XAI-based methods increase precision through better transparency and accountability that is incorporated in the proposed interactive 3D visualization structure. However, the vast majority of the existing XAI methods are based on single explanation methods and lack multi-level interpretability with both spatial and feature-level explanations.

### 2.6 Transformer-based and mamba-based architectures in lung cancer diagnosis

In recent years, Vision Transformers (ViT) and medical imaging variants have proven to be viable alternatives to CNN-based architectures. Swin Transformer (Liu et al., 2021) proposed the concept of hierarchical shifted window attention which allowed to process high-resolution medical images efficiently and obtain better results on 2D/3D classification tasks. Transformer-based models have showed competitive results in lung cancer diagnosis, where they can model long-range spatial dependencies which cannot be modelled by local convolutional kernels. This was extended to volumetric medical image analysis using MedFormer (Gao et al., 2022), which showed that transformer encoders can learn clinically meaningful representations from 3D CT without utilizing handcrafted features.

State-space models, especially Mamba variants (Gu and Dao, 2023), have recently been a major focus in medical imaging due to their linear complexity in the number of sequence elements, which overcomes the scalability challenge for attention-based transformers on high-resolution volumetric data. Vision Mamba (Zhu et al., 2024) has also shown performance comparable to Swin Transformer in medical image classification tasks while significantly reducing the computational requirements. TransUNet (Chen et al., 2021) is an intermediate solution that involves using transformer encoders and CNN decoders to benefit from both global attention and local feature extraction in a single framework. While these developments are promising, current transformer- or Mamba-based methods for lung cancer diagnosis often rely on either 2D slices or 3D volumes in isolation and lack an integrated incorporation of quantitative radiomic features and multi-level explainability within a single diagnostic workflow, the unique strengths of the proposed LungCraft framework.

Reviewed literature collectively identifies three gaps that have remained constant throughout and cannot be addressed by any single framework. First, hybrid volumetric-contextual deep learning approaches do not include quantitative radiomics at the feature-level but rather are based on purely learned representations lacking an interpretable morphological basis provided by handcrafted descriptors. Second, XAI-equipped frameworks use either spatial or feature-level explanation approaches, but not in combination, which prevents providing the clinician with a comprehensive diagnostic rationale. Thirdly, there is a lack of existing knowledge-graphs that integrate AI driven classification with interactive 3D tumour visualization in a clinician facing interface, creating a disconnect between computational prediction and clinically usable spatial information. LungCraft is specifically developed to fill all three gaps in an all-encompassing pipeline and the architectural and evaluation choices outlined in the next few sections are made with that in mind rather than with the goal of modifying existing architectures incrementally.

## 3 Proposed methodology

### 3.1 Overview

**Figure 1:**
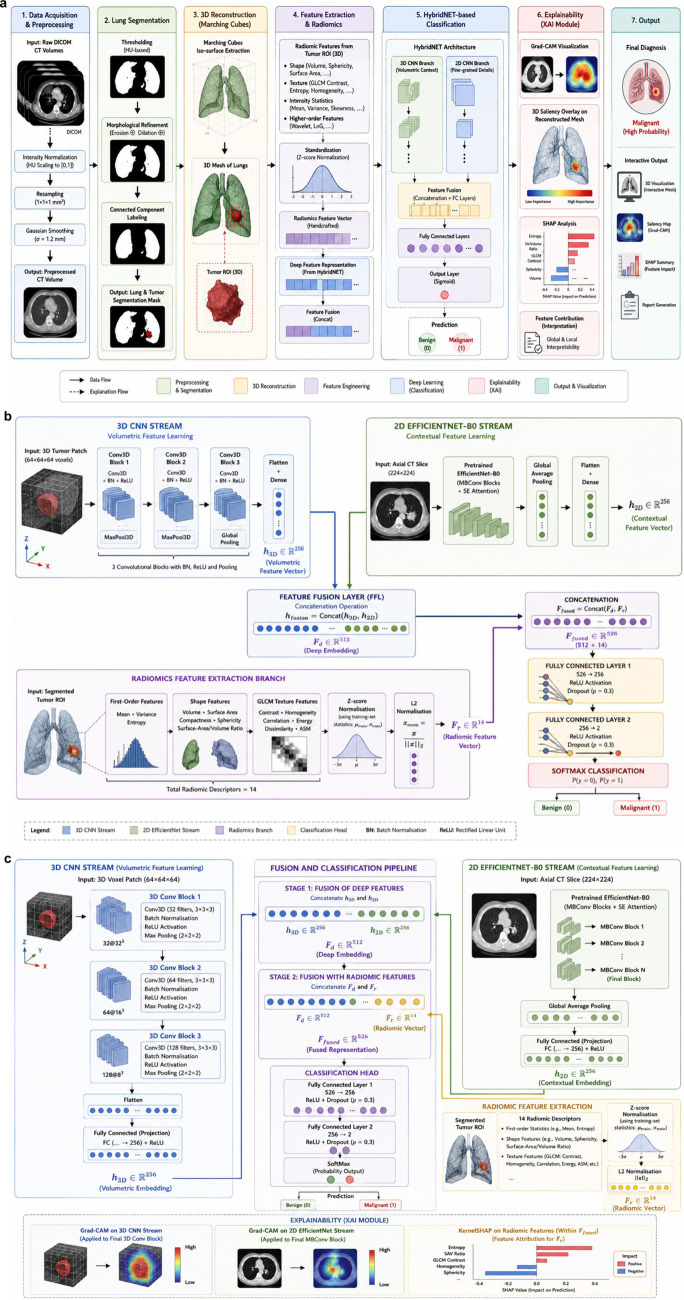
**(a)** LungCraft framework architecture illustrating the complete processing pipeline from raw DICOM input to classification output. **(b)** LungCraft feature fusion subprocess in detail. The 3D CNN stream takes 64 × 64 × 64 patches of voxels as input and passes it through three 3D convolutional blocks (with batch normalization and max pooling), which results in a 256-dimensional volumetric feature vector. **(c)** Detailed architectural diagram of HybridNET. The left branch shows the 3D CNN stream processing a 64 × 64 × 64 voxel patch through three progressively deeper convolutional blocks (32, 64, 128 filters) with batch normalization, ReLU activation and max pooling at each stage, followed by flattening and a fully connected projection to a 256-dimensional volumetric embedding h3D.

The proposed framework (Figure 1) is comprised of a novel architecture that integrates 3D medical imaging, radiomics-based quantitative analytics, hybrid deep learning and explainable artificial intelligence (XAI) methods to deliver high-accuracy prediction and transparent explanation of lung adenocarcinoma using CT scans. LungCraft uses a multi-stage, modular workflow that is designed to preserve volumetric fidelity and interpretability. Figure 1 shows the LungCraft framework architecture showing the entire framework, from raw DICOM input to the classification output. The pipeline consists of five steps: (1) preprocessing and lung segmentation; (2) 3D Marching Cubes reconstruction and radiomic feature extraction to obtain a 14-dimensional normalized feature vector F_r_; (3) dual stream HybridNET feature extraction, including a 3D CNN stream (256-dimensional volumetric embedding) and 2D CNN stream (256-dimensional contextual embedding) concatenated to form a 512 dimensional deep feature vector F_d_; (4) feature-level fusion by concatenating the F_d_ and F_r_ to generate a 526 dimensional fused representation F_fused_ followed by two fully connected layers; (5) SoftMax classification into benign and malignant categories, along with Grad-CAM spatial explanation and KernelSHAP radiomic feature attribution.

The preprocessing and normalization of CT scans is performed to standardize the voxel size and distribution of their intensities to initialize the framework. This process maintains consistency in the representation in various imaging modalities. Segmentation technique separates the parenchyma and tumour regions of the lungs after preprocessing with thresholding and morphological filtering to remove noise. These segmented areas are rebuilt into a high fidelity 3D model using the Marching Cubes algorithm. Marching Cubes algorithm produces smooth polygonal surfaces that preserve global morphology and local textural changes that are important in identifying lesions.

Radiomic analysis is performed on the reconstructed 3D structure in which handcrafted features like shape, sphericity, grey-level co-occurrence contrast and entropy are obtained to calculate tumour heterogeneity and boundary irregularities. These handcrafted features are standardized and combined with deep-learning representations created with the HybridNET model. The hybrid model applies the convolutional network which integrates the volumetric awareness of 3D CNNs and the contextual sensitivity of 2D CNNs. This hybrid model captures the coarse and fine-grained details which enable the model to distinguish between malignant and benign tumours with greater accuracy.

The predictions generated using the hybrid model are fed as input to Explainable AI (XAI) module to make sense of the predictions. Grad-CAM identifies regions of discrimination that exist within the region of interest (ROI) of the tumour that affect the prediction of the model. The method gives a visual description as colour-coded saliency overlay on 3D reconstructions. Then, SHAP (SHapley Additive exPlanations) algorithm is used to understand the contribution of the quantitative features of the radiomic features including entropy and surface-area-to-volume ratio to enhance the classification. These explanations enhances the interpretability of the proposed model LungCraft which in turn helps doctors make informed decisions when it comes to detecting and studying lung cancer.

Figure 1b shows LungCraft feature fusion subprocess in detail. The 3D CNN stream takes 64 × 64 × 64 patches of voxels as input and passes it through three 3D convolutional blocks (with batch normalization and max pooling), which results in a 256-dimensional volumetric feature vector. EfficientNet-B0 stream of the 2D EfficientNet-B0 generates a 256-dimensional contextual feature vector by taking 224 × 224 axial slices and processing them in pretrained convolutional layers. Concatenate the two vectors together to form the high dimensional (512) deep embedding F_d_. Using bottom branch, 14 radiomic descriptors are extracted, z-score normalized with training-set statistics, L2-normalised to obtain F_r_. In final fusion, the two fully connected layers (526 → 256 → 2) are followed by a ReLU and dropout (*p* = 0.3) and then softmax classification, using F_fused_ as the input vector, which is concatenated from F_d_ and F_r_.

### 3.2 Data acquisition and preprocessing

#### 3.2.1 Dataset description

The datasets of CT scans were acquired from the Cancer Imaging Archive (TCIA) that comprises 61 contrast-enhanced CT volumes of lung adenocarcinoma cases. The scans were saved in the DICOM format with the voxel size of 512×512×N where N denotes the number of axial slices.

#### 3.2.2 Intensity normalization

In order to address the issue of scanner variability and contrast differences, intensity normalization is done using the Hounsfield Unit (HU) range:


$$\begin{array}{l} I_{\mathrm{norm}}=\frac{I_{\mathrm{raw}}-HU_{\min}}{HU_{\max}-HU_{\min}}\;\text{where}\;HU_{\min}\\ =-1000\;\text{and}\;HU_{\max}=400\; \end{array}$$ (1)

Equation 1 converts voxel intensities into a range of [0,1] to improve contrast of tissues between air, soft tissue and bone materials.

#### 3.2.3 Resampling and noise reduction

To ensure isotropic resolution, the CT volumes are resampled to a uniform voxel spacing of 1×1×1 mm^3^. Gaussian smoothing is used in order to minimize the noise in the image:


$$\begin{array}{l} I_{\text{smooth}}(x,y,z)=\frac{1}{{\left(2\pi \sigma^{2}\right)}^{\frac{3}{2}}}\int I(x^{\prime },y^{\prime },z^{\prime })\\ \;\;\;\;\;\;\;\;\;\;\;\;\;\;\;\;\;\;\;\;\;e^{-\frac{{\left(x-x^{\prime }\right)}^{2}+{\left(y-y^{\prime }\right)}^{2}+{\left(z-z^{\prime }\right)}^{2}}{2\sigma^{2}}}dx^{\prime }dy^{\prime }dz^{\prime } \end{array}$$ (2)

High frequency noise due to scanner variability and resampling artefacts is suppressed by Gaussian smoothing. The kernel std. deviation was chosen to be σ=1.2 mm and the smoothing was performed in the isotropic voxel space after resampling to 1 × 1 × 1 mm^3^. This value was determined by performing a grid search over *σ* ∈ {0.8, 1.0, 1.2, 1.5, 2.0} mm on a held out set of five training volumes, where the criteria for choosing the value was the Dice Similarity Coefficient of the resulting lung segmentation mask with manual annotations, which, at the voxel level used, was highest for σ=1.2 mm (DSC 0.924) compared to σ=1.0 mm (DSC 0.918) and σ=1.5 mm (DSC 0.911). It was omitted to prevent data leakage, but the same value of σ=1.2 mm was used for all the datasets.

### 3.3 Lung segmentation

Segmentation separates the lung region, the background pixels and mediastinal structures using thresholding, morphological refinement and region-growing.

It is important to note here that these segmentation and classification modules in LungCraft are not performed simultaneously but are sequential. As explained in this section, a pre-processing step called Lung Segmentation is performed which separates the lung parenchyma and tumour area from other surrounding anatomical structures before feature extraction and classification. Segmentation does not share parameters with HybridNET nor is it trained simultaneously with the classification goal. The segmentation output is a binary mask that outlines the tumour ROI, which can be used for three purposes:

- It is used to segment the 64 × 64 × 64 voxel patch inputted to the 3D CNN stream of HybridNET,
- To segment axial slices, from which 224 × 224 inputs are cropped to the 2D CNN stream and
- To mask the volume on which radiomic features are calculated. These inputs, generated from the segmentation, are then classified independently by HybridNET.

The two modules are operationally linked by their input and output, but they are architecturally and parametrically independent and have completely different training processes.

#### 3.3.1 Thresholding

A binary mask created to improve thresholding is an HU-based mask:


$$M(x,y,z)=\{\begin{array}{l} 1,\;\text{if}-600<I_{\mathrm{norm}}(x,y,z)<-300\\ 0,\geq \;\text{otherwise} \end{array}$$ (3)

#### 3.3.2 Morphological operations

In order to eliminate small artifacts, erosion and dilation were used sequentially:


$$M^{\prime }=(M⊖B)⊕B$$ (4)

where *B* denotes a 3×3×3 structuring element. The two largest connected regions that represent the right and left lungs are retained with connected component labelling.

### 3.4 Marching cubes 3D reconstruction

Marching Cubes algorithm is applied to reconstruct 3D iso-surfaces of binary masks after segmentation to produce a polygonal mesh representation of the lung and tumour. An iso-surface is interpolated, on edges where intensity changes across the threshold value t between each voxel cube:


$$v_{i}=p_{1}+\frac{t-I(p_{1})}{I(p_{2})-I(p_{1})}(p_{2}-p_{1})$$ (5)

where $p_{1}$ and $p_{2}$ are the coordinates of the adjacent voxels and $v_{i}$ is the vertex of intersection. The result of equation 5 is used to construct the reconstructed surface S and smoothed with Laplacian filtering:


$$S^{\prime }(v)=v+\lambda \sum_{u\in N(v)}(u-v)$$ (6)

where N(v) are adjacent vertices and λ=0.2 is used to regulate the smoothing intensity. The result of this process is an interactive 3D model which can be visualized in the LungCraft Viewer that allows rotation, slicing and zoom to inspect the tumours in more detail.

### 3.5 Radiomic feature extraction

Radiomics consists of an extraction module which measures structural and textural heterogeneity in tumours based on CT scans. LungCraft computes three types of features, first-order statistical features, shape features and texture features.

#### 3.5.1 First-order statistical features

The voxel intensity distribution is represented in equation 7:


$$\text{Mean}=\frac{1}{N}\sum_{i=1}^{N}I_{i},\;\;\;\text{Entropy}=-\sum_{i=1}^{N}p(I_{i})\log\;p(I_{i})$$ (7)

where $p(I_{i})$ denotes the normalized histogram probability.

#### 3.5.2 Shape features

The shape characteristics are obtained based on the 3D mesh:


$$\text{Compactness}=\frac{V^{2}}{A^{3}},\;\;\text{Sphericity}=\frac{\pi^{1/3}{\left(6V\right)}^{2/3}}{A}$$ (8)

where are the tumour volume V and surface area A respectively. Lower values of compactness and sphericity are signs of non-regular tumour boundaries.

#### 3.5.3 Texture features

Grey-Level Co-occurrence Matrix (GLCM) features were used to measure the texture heterogeneity:


$$\text{Contrast}=\sum_{i,j}{\left(i-j\right)}^{2}P(i,j),\;\;\;\text{Homogeneity}=\sum_{i,j}\frac{P(i,j)}{1+|i-j|}$$ (9)

where P(i,j) indicates the joint probability of grey-level pairs that are separated by a predefined distance d .

#### 3.5.4 Feature normalization and fusion strategy

Before the fusion process, all the extracted radiomic features are standardized by using z-score normalization, as defined in Equation 10, which is computed only on the training set and applied to the validation and test sets to avoid distribution mismatch. The normalization process guarantees that the features obtained from the radiomic analysis and described with different scales and units, such as shape features, intensity features and GLCM texture features, are balanced in the fused representation and therefore do not overwhelm the learning signal.

The feature fusion approach works as follows. Let $F_{r}$ be the normalized radiomic feature vector, with m being the number of radiomic descriptors extracted. The present implementation is for m = 14, which includes three first-order statistical features (mean, variance and entropy), five shape features (volume, surface area, compactness, sphericity and surface-area-to-volume ratio) and six GLCM texture features (contrast, homogeneity, correlation, energy, dissimilarity and ASM). Let $F_{d}\in {\mathbb{R}}^{n}$ be the deep feature embedding of the penultimate fully connected layer of the HybridNET dual-stream model with n = 512 after the feature fusion layer as described in Equation 14. Before concatenation both Fr and Fd are L2-normalised to ensure that $F_{r}$ and $F_{d}$ contribute equally to the fused vector, regardless of differences in dimensionality of the embedding.

The radiomic features extracted were all normalized using the z-score standardization and then sent to the HybridNET model.


$$F_{\mathrm{norm}}=\frac{F-\mu_{F}}{\sigma_{F}}$$ (10)

where $\mu_{F}$ is the average and $\sigma_{F}$ standard deviation of the features computed during training. The same normalization parameters are applied to both validation and testing using equation 10 so that the results are consistent and there is no mismatch of distributions. After normalization, a feature-level fusion strategy is applied. Let $F_{r}\in {\mathbb{R}}^{m}$ be the normalized radiomic feature vector and $F_{d}\in {\mathbb{R}}^{n}$ indicate deep feature embeddings which are extracted from the unified architecture.

The fused feature representation is achieved by simply concatenating the features:


$$F_{\text{fused}}=[F_{d}F_{r}]\in {\mathbb{R}}^{n+m}$$ (11)

where $F_{d}$ indicates deep features obtained from the final convolutional layers of the hybrid 3D-2D network which are flattened prior to fusion. The resulting fused vector $F_{\text{fused}}\in {\mathbb{R}}^{m+n}$ is obtained using Equation 11 is passed through the FCL for final classification. This will generate a fused vector with a dimensionality of 512 + 14 = 526. After this concatenated vector, it is fed to two fully connected layers with ReLU and dropout regularization (*p* = 0.3) and then to a SoftMax classification layer. The first fully connected layer maps $F_{\text{fused}}$ from 526 dimensions to 256 dimensions and the second to 2 dimensions representing the two output classes (benign and malignant). This dimensionality reduction is explicit and makes the network learn an embedding of low dimensionality that combines spatial deep features and hand-designed radiomic descriptors.

The fusion is done at the feature level, instead of the decision level (i.e., fusion of radiomic features and deep features prior to the final classification, rather than fusion of individual outputs from classifiers) and allows the final fully connected layers to learn cross-modal interactions between deep spatial representations and radiomic descriptors. This differs from late fusion or ensemble approaches, where each modality makes its own prediction, which is then combined. The feature-level fusion strategy thus enables the model to make use of complementary information between the irregular boundary descriptors learned via radiomics and the hierarchical spatial patterns learned via the convolutional streams, instead of regarding them as independent classification signals. The combination allows the model to utilize both handcrafted radiomics features and deep learned features. The radiomics descriptors measures tumour heterogeneity and morphological structure and the spatial hierarchy and contextual patterns are measured by the deep features. The fusion is performed following normalization to equalize the contribution of features and avoid dominance of high magnitude features to enhance predictive performance and explainability.

Figure 1c shows the HybridNET’s detailed architecture. The left stream is the 3D CNN, in which the 64 × 64 × 64 voxel patch is fed through three successive deep convolutional blocks (32, 64, 128 filters) with batch normalization, ReLU activation and max pooling after each block and finally flattened and projected to h3D, a 256-dimensional volumetric embedding. The right branch represents the 2D EfficientNet-B0 stream processing an axial slice of size 224 × 224 from the pretrained MBConv blocks and global average pooling, which is then followed by a fully connected projection to a 256-dimensional contextual embedding h2D. The centre column depicts the sequential fusion operations: h3D and h2D are first concatenated to create the 512-dimensional deep embedding Fd, then Fd is concatenated with the 14-dimensional radiomic vector Fr creating the 526 dimensional fused representation Ffused, which is finally passed through the two layer classification head (526 → 256 → 2) using ReLU, dropout and SoftMax to generate a benign or malignant prediction. Grad-CAM is used to create spatial heatmaps for the final 3D convolutional block and final 2D EfficientNet block and KernelSHAP is used to create radiomic feature attributions for Fr within Ffused.

### 3.6 HybridNET deep learning architecture

The HybridNET classifier is a hybrid network that consists of 3D-CNN and 2D-CNN modules that learns both spatial and contextual information.

#### 3.6.1 Architecture description

The LungCraft architecture consists of 3D Stream that is used for volumetric processing of medical data that involves the acquisition of spatial relationships across several slices of a CT scan. It works on 3D voxel cubes 64×64×64 by excising the volume that exists around the annotated tumour. This effectively captures the volumetric context and morphological continuity of lesions that are usually lost in 2D slice-based analysis. Each axial slice is resized to 224 × 224 pixels and passed to the 2D convolutional neural network backbone. As a result, all experiments reported in this manuscript used EfficientNet-B0 as the 2D backbone, because of the favorable balance between parameter efficiency (5.3 M parameters) and feature extraction capability compared to ResNet50 (23.1 M parameters), which mitigates the risk of overfitting on the small primary cohort. Table 1 is an independent evaluation of the ResNet50 as a standalone 2D baseline and should not be interpreted as evidence of its performance as a candidate backbone in the architecture design process, the HybridNET architecture only utilizes EfficientNet-B0 as the 2D stream component. The input voxel cube is passed through a series of 3D convolutional layers. Then, it is preceded by batch normalization, ReLU activation and 3D max-pooling. These layers sequentially encode spatial hierarchies and depth-wise associations, allowing the model to acquire structural hints like discontinuous tumour edges, internal heterogeneity and variations in the surrounding tissue areas. The convolutional kernel size is set to 3×3×3 which is optimized to extract fine-grained features with low computational cost.


$$h_{3D}=f_{3}(W_{3D}*X_{3D}+b_{3D})$$ (12)

where ∗ is 3D convolution, $f_{3}$ denotes ReLU activation and the learnable parameters are denoted as $W_{3D}$ and $b_{3D}$ .

**Table 1:** Comparative performance of baseline and proposed HybridNET architectures for lung cancer classification.

Model	Accuracy (%)	Precision (%)	Recall (%)	F1-score (%)	AUC	Parameters (M)
2D CNN (baseline)	78.6 ± 1.9	76.4 ± 2.1	77.2 ± 2.0	76.8 ± 2.0	0.82 ± 0.02	12.3
3D CNN (baseline)	82.4 ± 1.6	80.1 ± 1.8	81.3 ± 1.7	80.7 ± 1.7	0.85 ± 0.02	16.8
ResNet50 (2D)	84.7 ± 1.4	83.5 ± 1.6	82.9 ± 1.5	83.2 ± 1.5	0.87 ± 0.02	23.1
DenseNet121 (2D)	85.3 ± 1.3	84.1 ± 1.5	83.8 ± 1.4	83.9 ± 1.4	0.88 ± 0.01	21.7
InceptionV3 (2D)	87.0 ± 1.2	86.5 ± 1.3	85.9 ± 1.3	86.2 ± 1.3	0.89 ± 0.01	25.4
EfficientNet-B0 (2D)	88.1 ± 1.1	87.6 ± 1.2	86.9 ± 1.2	87.2 ± 1.2	0.90 ± 0.01	17.8
HybridNET (Proposed)	91.3 ± 0.9	90.6 ± 1.0	89.8 ± 1.0	90.2 ± 1.0	0.93 ± 0.01	19.6

The LungCraft architecture’s 2D stream is aimed at detecting fine-grained textural and morphological patterns in each axial CT slice. This stream, in contrast to the volumetric 3D stream, represents spatial continuity between slices, highlights in-plane structural variations. These structural changes are edge sharpness, pixel intensity gradient and tumour boundary irregularities that are important features of malignancy.

Individual axial slices are re-sized to 224×244 pixels and fed through a 2D convolutional neural network (CNN) backbone (e.g., EfficientNet-B0 or ResNet50) which are trained on large medical datasets in advance. The convolutional layers obtain hierarchical texture features, beginning with the low-level features such as edges and gradients to the high-level semantic features such as lesion shape and density contrast.


$$h_{2D}=f_{2}(W_{2D}*X_{2D}+b_{2D})$$ (13)

The LungCraft framework consists of Feature Fusion Layer (FFL) which is an integration that combines the complementary capabilities of the 2D texture-based stream and the 3D volumetric stream into one representation to be used in medical diagnosis. The combination of the two allows the model to take advantage of both the local 2D radiographic information and the global 3D spatial information, so that both fine-grained and volumetric information is used in the final classification. Both streams produce flattened feature vectors which are 2D CNN and 3D CNN branch outputs which are concatenated to create a hybrid feature embedding and then a fully connected layer and dropout regularization (p=0.3) .


$$h_{\text{fusion}}=\text{Concat}(h_{3D},h_{2D})$$ (14)

The proposed architecture performs the final classification and probability estimation of lung lesions. It determines whether it is benign or malignant based on the integrated multi-stream feature representation. The FFL produces a combined hybrid feature vector F passed through one or more fully connected (dense) layers with ReLU activation to learn non-linear correlations between the learned features. The sequential integration is explained as follows: the 3D CNN stream is used to generate a spatial feature vector (flattened to 256 dimensions), the 2D CNN stream to obtain a contextual feature vector (flattened to 256 dimensions), the 256 spatial and 256 contextual feature vectors are concatenated into a 512-dimensional deep feature embedding ( $F_{d}$ ) as defined in Equation 14 and $F_{d}$ is in turn concatenated with the 14-dimensional normalized radiomic vector ( $F_{r}$ ) to create the 526-dimensional fused representation ( $F_{\text{fused}}$ ) that is fed to the final classification layers and thus volumetric, contextual and handcrafted radiomic information are all represented in the last decision boundary.

The final dense layer generates a two-dimensional vector that provides logit scores for each class. The logits are transformed to normalized class probabilities using SoftMax activation function where k=2 corresponds to benign and malignant tumour categories.


$$P(y=k|x)=\frac{e^{z_{k}}}{\sum_{i=1}^{K}e^{z_{i}}}$$ (15)

In the case of HybridNET architecture, the 2D branch is trained with pretrained weights on ImageNet dataset to take advantage of transfer learning to enhance the effectiveness of feature extraction. The pretraining facilitates the network to acquire the low- and mid-level generic features to improve the convergence rate and the generalization process (Deng et al., 2009; Krizhevsky et al., 2012). He (Kaiming) deep network-appropriate initialization was used to initialize the 3D branch. It employs ReLU activation function to make the gradient propagation stable throughout the training process (He et al., 2015). Nonetheless, the pretraining was not applied to the 3D branch and it was trained randomly since there are restrictions in the availability of large scale annotated 3D medical datasets. During the training process, pretrained 2D layers were fine-tuned while 3D branch parameters were learned entirely from the target dataset. to learn completely on the target dataset. This is a hybridized initialization strategy that enhances the stability of convergence, it facilitates successful acquisition of volumetric and cross-sectional representations.

#### 3.6.2 Training and optimization

The LungCraft model uses Categorical Cross-Entropy (CCE) as its loss function to maximize the classification performance of the HybridNET model. This loss term is common in multi-class or binary classification tasks where the model provides probability distributions for discrete categories. CCE is used to measure the difference between the estimated probability distribution of the SoftMax layer and the actual class labels. Training aims at reducing this loss, hence increasing the chances of making the right classification. Mathematically it can be expressed as:


$$L=-\sum_{i=1}^{N}y_{i}\;\log(\hat{y_{i}})$$ (16)

where $y_{i}$ is the ground truth and $\hat{y_{i}}$ is the predicted probability. For each input CT scan, the loss imposes penalty on wrong predictions based on their confidence. During backpropagation, the loss function gradients are calculated and updates the network weights to minimize errors in prediction at each iteration. Categorical Cross-Entropy loss can guarantee a stable and rapid convergence because it allows the model to provide probabilities that are highly consistent with the actual class distributions.

The training process uses Adam optimizer, a stochastic gradient-based optimization algorithm where learning rate is set as $\eta =10^{-3}$ , $\beta_{1}=0.9$ and $\beta_{2}=0.999$ . It combines the advantages of both AdaGrad and RMSProp. The Adam optimizer varies the learning rate of each parameter based on the first and second result of the gradients. At the early stopping checkpoint, the model had a training accuracy of 92% and validation accuracy of 80% (which measures the validation accuracy in the model during the training process and does not indicate the final post-training accuracy). This test accuracy of 91.3% is reported in Table 1 and is the accuracy of the inference with this retained checkpoint on the held-out test partition after all training has finished, without updating any parameters (Alakwaa et al., 2017; Wu et al., 2023). Both figures are from different evaluation stages on different data partitions and are not directly comparable. The performance of the training, validation and test sets in a single table is shown in Table 2. We explicitly recognize that the 12-percentage-point difference between training and validation accuracy, along with the 19.6 million sizes of the parameters in a primary cohort of about 43 training patients, poses a material risk of overfitting. While this risk is partially mitigated through regularization measures (dropout is set at 0.5, *λ* is 1 × 10^−4^, patience is 10 epochs and data augmentation is used), it cannot be completely removed at this scale of data and all performance numbers mentioned throughout the paper should be read within this context. In order to avoid model overfitting and to enhance generalization, several regularization methods were used in the training process. The FCLs with dropout rate (0.5) were used in the dropout strategy to minimize neuron co-adaptation. Moreover, L2 regularization (weight decay) was added with a coefficient of $1\times 10^{-4}$ to limit model complexity and stabilize learning.

**Table 2:** Extended comparison of HybridNET against state-of-the-art transformer-based and mamba-based architectures.

Model	Architecture type	Accuracy (%)	Precision (%)	Recall (%)	F1-Score (%)	AUC	Parameters (M)
Swin transformer	Transformer (2D)	89.4 ± 1.0	88.7 ± 1.1	88.1 ± 1.1	88.4 ± 1.1	0.92 ± 0.01	28.3
Vision Mamba	State-space (2D)	88.9 ± 1.1	88.2 ± 1.2	87.6 ± 1.2	87.9 ± 1.2	0.91 ± 0.01	22.1
MedFormer	Transformer (3D)	88.1 ± 1.1	87.4 ± 1.2	86.8 ± 1.2	87.1 ± 1.2	0.90 ± 0.01	31.4
TransUNet	Hybrid CNN-Transformer	87.6 ± 1.2	86.9 ± 1.3	86.2 ± 1.3	86.5 ± 1.3	0.90 ± 0.01	29.7
HybridNET (Proposed)	Hybrid 3D-2D CNN + Radiomics	91.3 ± 0.9	90.6 ± 1.0	89.8 ± 1.0	90.2 ± 1.0	0.93 ± 0.01	19.6

Each model was tested in the same way, using the same splits of the dataset, the same preprocessing approach, the same optimizer, learning rate, batch size and augmentation scheme as outlined in Section 4.2. Both Swin Transformer and Vision Mamba models were used in 2D mode, and the patient level majority voting aggregation method was used. Thereafter, MedFormer was used in 3D volumetric mode on 64 × 64 × 64 patches as per the HybridNET 3D stream input. The segmentation decoder has been removed, and a fully connected classification head has been added to TransUNet to enable binary classification. The weights of all pretrained models were also obtained from publicly available repositories and fine-tuned on the TCIA training split in the same conditions as HybridNET.

The data augmentation methods encompass random flipping, rotation (±15), scaling and elastic deformation were only used on the training set to make the data robust. Early stopping criteria was performed using validation loss to avoid overfitting. Training was terminated if the validation loss did not improve for 10 consecutive epochs (patience=10) . The model with the best validation performance was retained for final evaluation. The strategy makes sure that the model is generalized optimally without having to unnecessarily repeat training. Scheduling of learning rate was also used to slow down the learning rate when plateau in validation loss occurred and further stabilize the training process.

##### 3.6.2.1 HybridNET architecture specification

For full reproducibility the HybridNET architecture is detailed in Table HN1 layer by layer. It consists of three parts - 3D CNN stream, 2D EfficientNet-B0 stream and fusion and classification head. The details of HybridNET’s layer-by-layer architecture specification are shown in Table 3.

- Input: 3D CNN Stream is a voxel patch of size 64 × 64 × 64 × 1 from a segmented tumour region. Each stream consists of three convolutional blocks, each of which is composed of a 3D convolutional layer (kernel size 3 × 3 × 3, padding 1), batch normalization, ReLU activation and 3D max pooling (kernel size 2 × 2 × 2, stride 2). Each block has progressively doubled filter counts, e.g., 32, 64 and 128 in blocks 1, 2 and 3 respectively, to represent spatial hierarchies. After three pooling operations, the spatial dimensions are compressed to 8 × 8 × 8 and the 128 × 8 × 8 × 8 feature map is flattened and mapped to a 256-dimensional volumetric embedding h3D ∈ ℝ^256^ by a fully connected layer.
- 2D EfficientNet-B0 Stream: Input: 224 × 224 × 3 is an axial slice from the segmented tumour region duplicated across three channels to meet the EfficientNet-B0 input convention. The following set of convolutional layers are pre-trained using ImageNet weights and fine-tuned during training. A global average pooling layer is used after the last MBConv block to create a 1,280-dimensional feature vector that is mapped to a 256-dimensional contextual embedding h2D ∈ ℝ^256^ by a fully connected layer with ReLu activation.
- Fusion and Classification Head: h3D and h2D are fused to form 512 dimensional deep embedding F_d_. The 14-dimensional normalized radiomic vector F_r_ is concatenated with F_d_ to get the fused representation F_fused_ of 526 dimensions. F_fused_ is sent to two fully connected layers that project from 526 to 256 dimensions with a ReLU activation and dropout (*p* = 0.3) and then from 256 to 2 dimensions. The binary class probabilities (benign/malignant) are obtained using SoftMax activation.

**Table 3:** HybridNET layer-by-layer architecture specification.

Stream	Layer	Configuration	Output shape	Parameters
3D CNN	Input	-	64 × 64 × 64 × 1	-
	Conv3D Block 1	32 filters, 3 × 3 × 3, pad = 1, BN, ReLU, MaxPool 2 × 2 × 2	64 × 64 × 64 × 1	896
	Conv3D Block 2	64 filters, 3 × 3 × 3, pad = 1, BN, ReLU, MaxPool 2 × 2 × 2	16 × 16 × 16 × 64	55,360
	Conv3D Block 3	128 filters, 3 × 3 × 3, pad = 1, BN, ReLU, MaxPool 2 × 2 × 2	8 × 8 × 8 × 128	221,440
	Flatten	-	65,536	-
	FC + ReLU	256 units	256	16,777,472
2D EfficientNet-B0	Input	-	224 × 224 × 3	-
	MBConv Blocks	Pretrained EfficientNet-B0	7 × 7 × 1,280	4,007,548
	Global Avg Pool	-	1,280	-
	FC + ReLU	256 units	256	4,007,548
Fusion head	Concatenate (h3D, h2D)	-	512	-
	Concatenate (Fd, Fr)	14 radiomic features	526	-
	FC + ReLU + Dropout(0.3)	256 units	256	134,912
	FC + SoftMax	2 units	2	514

This table shows, batch normalization (BN), fully connected layer (FCL), Mobile inverted bottleneck convolution (MBConv), numbers of parameters are only approximate and include all EfficientNet-B0 pretrained parameters. The 3D FC layer takes up most parameters as it has a large flattened spatial dimension (65,536) before it is projected. Fr is normalized radiomic feature vector (14 features), Ffused dimensionality is 526, there have been no dropouts, only during training. It is important to note that the EfficientNet-B0 (2D) in this table is a standalone 2D classifier applied to the axial slices and should not be confused with the EfficientNet-B0 backbone used inside the HybridNET 2D stream. HybridNET is a combination of EfficientNet-B0 2D feature extraction, a 3D convolutional stream and radiomic feature fusion while the standalone EfficientNet-B0 baseline only involves the 2D feature extraction.

### 3.7 Explainable AI integration

In order to achieve clinical transparency, LungCraft uses two complementary interpretability mechanisms Grad-CAM and SHAP.

#### 3.7.1 Gradient-weighted class activation mapping (grad-CAM)

Grad-CAM visualizes the feature maps obtained in convolutional layers and its contribution to the classification outcome (Kumaran et al., 2024; Hammad et al., 2025). The gradient-based importance weights of a target class 𝑐 and feature map activations 𝐴_𝑘_ are:


$$\alpha_{k}^{c}=\frac{1}{Z}\sum_{i}\sum_{j}\frac{\partial y^{c}}{\partial A_{\mathrm{ij}}^{k}}$$ (17)

where 𝑍 is the number of pixels. The class activation map is computed as:


$$L_{\text{Grad}-\mathrm{CAM}}^{c}=\text{ReLU}(\sum_{k}\alpha_{k}^{c}A^{k})$$ (18)

These heatmaps are superimposed on 3D tumour reconstruction, which allows decision regions to be visually verified.

#### 3.7.2 SHapley additive exPlanations (SHAP)

SHAP explains model results through the estimation of the marginal contribution of each feature according to cooperative game theory proposed by Fontes et al. (2024). SHAP provides explanations for the model predictions by estimating the contributions of each feature based on cooperative game theory. In this study, the SHAP variant is KernelSHAP which is model agnostic and can be used for the fused radiomic feature vector that is fed into the final fully connected classification layers of HybridNET. Explanation module was implemented using KernelSHAP as it was selected over TreeSHAP and DeepSHAP since the radiomic feature that is input to the explanation module is a structured tabular vector, not raw image data and KernelSHAP can operate on this representation without having access to internal neural network gradients or tree structures (Lundberg and Lee, 2017). Similar to common usage of KernelSHAP approximation, the explainer was configured on a background dataset of 50 training instances sampled randomly and SHAP values were calculated for all patients in the test-set.

The SHAP value of feature i of a prediction function f(x) is obtained.


$$\phi_{i}=\sum_{S\subseteq F\{i\}}\frac{|S|!(|F|-|S|-1)!}{|F|!}[f(S\cup \{i\})-f(S)]$$ (19)

where F is the feature set. In the current implementation, the feature set F is the radiomic feature vector Fnorm per patient, which contains first order statistical, shape and GLCM texture features, as normalised in Equation 10. SHAP values were calculated using the KernelSHAP approximation with settings nsamples = 1,000 to balance the approximation quality and compute time. The positive SHAP values are those that are associated with the classification of malignancy (high entropy, irregular boundary) whereas the negative values are those that are associated with benign tumours (homogeneity). Grad-CAM (spatial) and SHAP (feature-level) interpretability can be combined to offer local and global explainability in a variety of cases provided by the proposed model.

#### 3.7.3 Interpretability index scoring methodology

The Interpretability Index listed in Table 4 is an expert evaluation score that is structured along a five-point Likert scale (1 = not interpretable, 5 = fully interpretable) to be used as a comparative measure of clinical transparency for the various models. Two blind reviewers experienced in medical imaging and not involved in the development of the LungCraft framework scored them.

**Table 4:** Segmentation performance comparison of LungCraft and baseline architectures.

Segmentation method	DSC	IoU	Sensitivity	Specificity
Threshold + Region growing	0.872 ± 0.024	0.811 ± 0.028	0.857 ± 0.026	0.889 ± 0.022
2D U-Net	0.903 ± 0.019	0.851 ± 0.022	0.894 ± 0.021	0.913 ± 0.018
3D V-Net	0.917 ± 0.016	0.864 ± 0.019	0.903 ± 0.018	0.924 ± 0.015
Proposed hybrid segmentation (LungCraft)	0.926 ± 0.014	0.879 ± 0.017	0.912 ± 0.016	0.931 ± 0.013

Each evaluator assessed the models individually based on five pre-defined criteria, each having equal weight:

- Spatial localization - whether the model identifies the tumour region in a manner consistent with radiological expectation.
- Feature transparency - whether the model provides a meaningful indication of which imaging features influenced the classification decision.
- Explanation modality - whether the explanation output is presented in a format accessible to a clinical user.
- Consistency - whether explanations remain reasonably stable across cases with comparable presentations.
- Clinical alignment - whether highlighted regions and attributed features correspond to recognized radiological indicators such as irregular margins and heterogeneous texture.

Each criterion was scored independently by each reviewer, on a scale of 1–5. The final Interpretability Index for each model is the average for each of the five criteria and the two reviewers. The clinical relevance of the Grad-CAM was reviewed by two raters and the interrater agreement was calculated as Cohen’s kappa with a moderate agreement of 0.45 (n = 6 cases) and a raw observed agreement of 83.3% (Landis and Koch, 1977). The ICC of 0.79 reported in Section 3.7.3 is a different measure of agreement over the five structured criteria used on all the models in Table 4 and should not be compared to the kappa computed for the binary relevance rating that was based on the results of the grad-CAM approach. For models that did not return an explainability output, the score for feature transparency was set to 1 and the score for explanation modality was set to 1, both by default (no explainable output). We recognize that for models coming from previous literature, the scoring was also based on the explanation output described and illustrated in the publications and that these scores should only be viewed as an indication (Table 5).

**Table 5:** Disaggregated classification performance of HybridNET across internal and external test sets (Mean ± 95% CI). 95% CIs estimated via non-parametric bootstrapping (10,000 resamples) on patient-level predictions.

Test set	*n* (patients)	Accuracy (%)	Precision (%)	Recall (%)	F1-Score (%)	AUC
TCIA (internal)	~9	91.3 (83.4–97.2)	89.6 (87.8–91.3)	89.8 (81.3–96.1)	90.2 (81.7–96.4)	0.93 (0.84–0.99)
LIDC-IDRI (external)	1,018	88.7 (87.3–90.1)	87.9 (86.4–89.3)	86.5 (85.0–88.0)	87.2 (85.7–88.6)	0.91 (0.90–0.92)
NSCLC-radiomics (external)	422	89.6 (87.8–91.3)	88.8 (87.0–90.5)	87.9 (86.0–89.7)	88.3 (86.5–90.1)	0.92 (0.90–0.93)

TCIA CIs are wide reflecting the ~9-patient internal test partition; external CIs are narrow owing to the substantially larger cohort sizes. This table shows training and validation results refer to the performance of the model on an epoch up to the chosen epoch that has the best validation loss. Test figures are inference on the held-out partition following training; values in parentheses are 95% confidence intervals derived by non-parametric bootstrapping (10,000 resamples) at the patient level. The validation accuracy is a result of monitoring during training and should not be compared to the evaluation in testing.

## 4 Results

### 4.1 Dataset description and preparation

The main dataset to be evaluated is The Cancer Imaging Archive (TCIA) that comprises 61 contrast-enhanced CT volumes of patients with lung adenocarcinoma with annotations. The CT volumes were acquired with a thickness of 1.0 to 2.5 mm. in mediastinal and lung windows. The masks (ground truth) were annotated manually. Experiments were carried out on the LIDC-IDRI (Lung Image Database Consortium) dataset (Armato et al., 2011) to test its performance under various conditions. It is comprised of 1,018 CT images that have annotations of the nodules by radiologists. Lastly, the NSCLC-Radiomics data (TCIA subset) (Clark et al., 2013) is a dataset of 422 patients with mask segmentations and labels. The TCIA dataset was used exclusively for model training and internal validation. The TCIA dataset was employed only as a model training and internal validation set. The LIDC-IDRI and NSCLC-Radiomics datasets were held-out external test sets and no samples from either of these datasets were used for training, validation, or hyperparameter optimization in any form. No external cohorts were used to assess any of the model design decisions.

**Figure 2:**
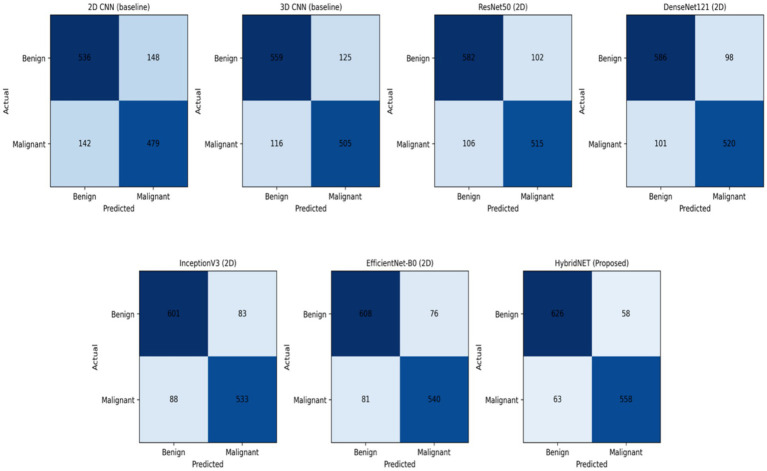
Confusion matrices for baseline models and the proposed HybridNET model. Counts reflect slice-level predictions on the test partition (approximately 1,305 slices derived from the 15% patient-level split of the TCIA cohort). Performance metrics reported in Table 1 are computed at the patient level via majority voting across per-patient slices and should not be directly compared to slice-level counts shown here. Slice-level matrices are presented for illustrative purposes only.

The slices were obtained by preprocessing all the CT volumes of the considered datasets. The CT volumes were split into several axial slices, which were extracted after being resampled to an isotropic resolution $\left(1\times 1\times 1\;mm^{3}\right)$ and scaled to the range of [−1000,400] Hounsfield Unit (HU). Only slices with relevant lung regions and annotated tumour areas were retained to be analysed leaving a total of about 8,700 slices in all datasets. Notably, data partitioning was conducted at the patient level in a strict manner before extracting the slices. Each slice of a particular patient was placed in only one subset (training, validation or testing) so that there was no overlap of slices of the same patient within different splits. It should be noted that data partitioning is done at patient level, but each confusion matrix in Figure 2 is based on the individual CT slice, not the patient. The total slice pool (~8,700 slices from all datasets) was split based on the patient-level split, resulting in ~6,090 training slices, ~1,305 validation slices and ~1,305 test slices. Thus, the counts in Figure 2 reflect the predictions at a slice level and this is important in the light of the discussion on interpretation of reported performance metrics as discussed in Section 4.3. The training process did not involve combining the datasets to avoid data leakage and to remove bias. This patient-based partitioning is in line with best practices in medical imaging to guarantee credible model assessment.

**Figure 3:**
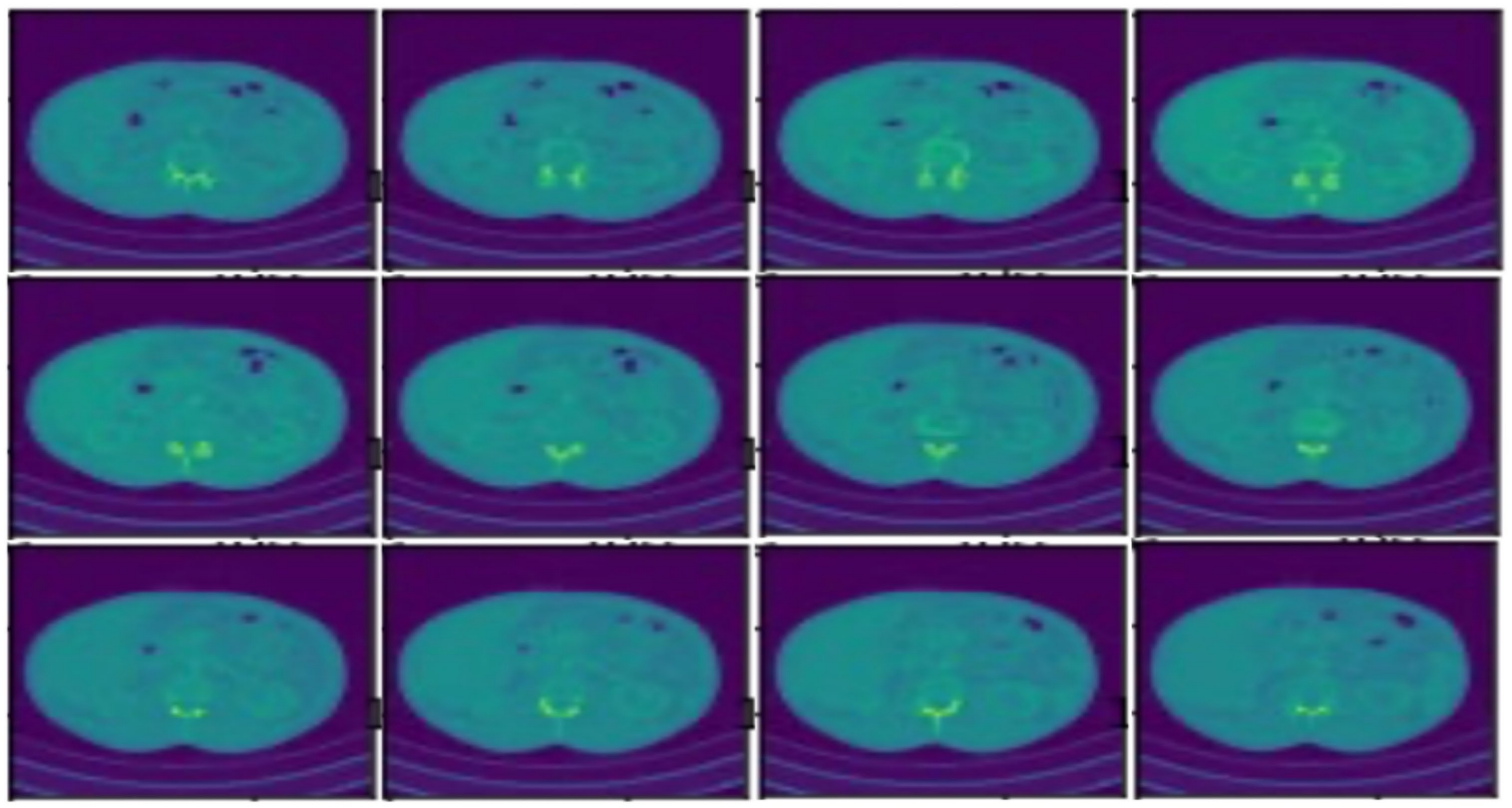
Data before applying preprocessing techniques.

**Figure 4:**
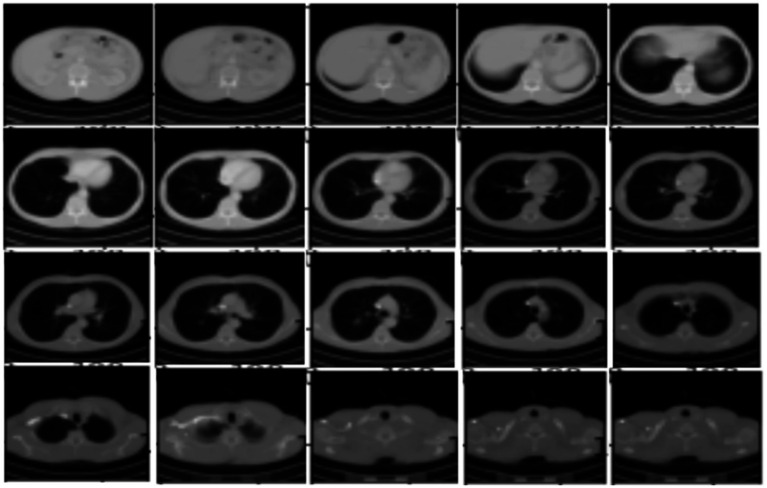
Data after applying preprocessing techniques.

Data partitioning was done at the patient level to make sure that the slices of the same patient are not shared between training, validation and testing. The data was categorized into training (70%), validation (15%) and testing (15%). It should be noted that this division results in ~43 training, ~9 validation and ~9 test patients in the primary TCIA cohort. With this modest internal test size, the internal test metrics reported in the tables should be interpreted with care; the 95% confidence intervals of all internal test classification metrics in Table 1 were obtained using non-parametric bootstrapping on the patient-level predictions (10,000 resamples) and generalizability is primarily evaluated on the external test sets, which provide significantly larger and more diverse evaluation cohorts. The loss functions such as Binary Cross-Entropy (BCE) and Dice/IoU loss were combined to maximize the performance of segmentation and enhance the delineation of boundaries. Figure 3 shows the raw input data, which consists of raw CT slices with variable intensity distributions, slice-to-slice noise and intensity. These problems have the potential to obscure finer anatomical features and influence the accuracy of segmentation and feature extraction. Figure 4 demonstrates the processed data after intensity normalization, Gaussian denoising, histogram equalization and standardization. The increased contrast and homogenous intensity range enables pulmonary structures, nodules and tumour boundaries to be more prominent. This standardization guarantees similar voxel intensity representation in all scans that can be used to compute radiomic and converge a model.

### 4.2 Implementation details

The LungCraft framework was written in Python 3.11, with the use of TensorFlow 2.15 and Keras 3.0 as the main deep learning platforms to develop and train the models. Quantitative extraction of radiomic features like shape, texture and intensity descriptors was performed using the PyRadiomics v3.1.0 library, whereas high-fidelity 3D reconstruction, visualization and rendering of lung tumour models was performed using the VTK, PyVista and Open3D libraries. SHAP explanations were generated using the KernelSHAP explainer from the SHAP python library (version 0.42.1), via the normalised radiomic feature vector, with a background reference dataset of 50 randomly sampled training instances (nsamples = 1,000). The experiments were performed on a high-performance workstation with an NVIDIA RTX A5000 (24 GB VRAM) graphics card, an Intel Xeon Silver 4,316 (2.30 GHz) processor, 256 GB RAM and Ubuntu 22.04 LTS operating system, which is optimally configured to have a high level of computation. The training setup used Adam optimizer with learning rate of $1\times 10^{-3}$ and momentum ( $\beta_{1}=0.9$ 0 and $\beta_{2}=0.999$ 0) which provided stable gradient updates. The model was trained over 120 epochs with a batch size of 8 using dropout regularization (p=0.3) and L2 weight decay $\left(\lambda =1\times 10^{-4}\right)$ to avoid overfitting.

Categorical cross-entropy loss was used in binary classification and early stopping with a threshold of 10 epochs was used to avoid overtraining after the validation loss levelled. To make the HybridNET model stable in terms of convergence and gradient flow, He-normal weight initialization of convolutional layers and Xavier weight initialization of dense layers were used to initialize the model. The whole training pipeline was to be accelerated using a mix of floating point (float16) on a GPU because it could achieve the highest possible training throughput and consume less memory without accuracy loss. The Gaussian smoothing parameter σ=1.2 mm was chosen using segmentation DSC as a criterion on 5 held-out training volumes and was kept constant before evaluation on validation or test volumes, as described in Section 3.2.3.

**Figure 5:**
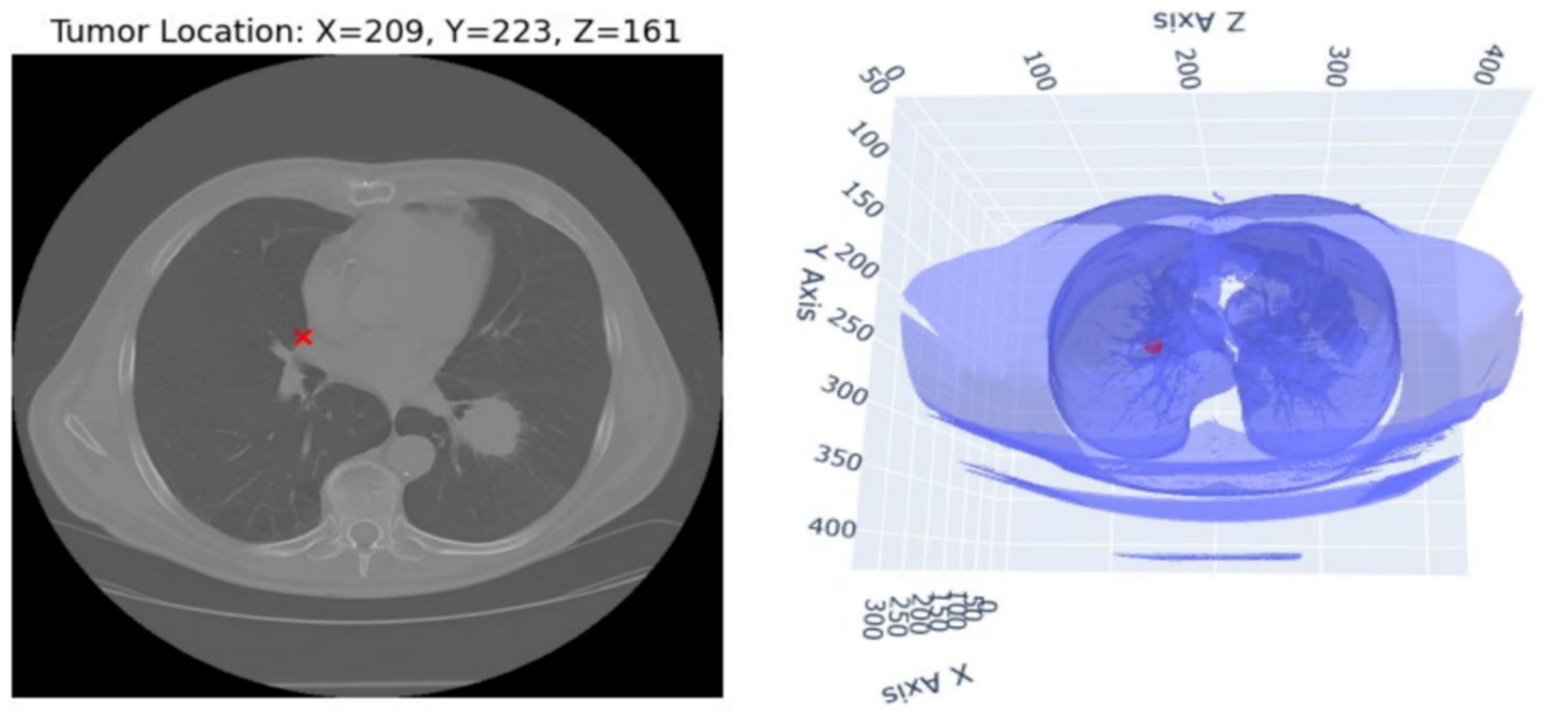
Tumour localization and 3D reconstruction from NSCLC-Radiomics dataset.

Figure 5 shows the localization and 3D volumetric reconstruction procedure of tumours used in LungCraft framework. The left panel depicts an axial CT slice of the NSCLC-Radiomics dataset in 2D with the detected tumour area being identified by a red cross at the coordinates (X = 209, Y = 223, Z = 161). The lesion is found in the area of the mediastinal region and the exact annotation is used to initialize the seed-point of the segmentation correctly. The right panel shows the equivalent 3D reconstructed lung volume, in which the tumour is seen in the context of the anatomy of the thoracic cavity. VTK and PyVista libraries were used to produce the volumetric rendering by stacking 2D slices to create a high-fidelity 3D mesh to be used in spatial analysis.

For fair comparison, all the baseline models (2D CNN, 3D CNN, ResNet50, DenseNet121, InceptionV3 and EfficientNet-B0) have been re-implemented and trained on the same experimental conditions on the same dataset splits, preprocessing pipeline and evaluation protocol as the proposed HybridNET model. We note that when EfficientNet-B0 is cited alone for a baseline in Table 1, it is the 2D-only EfficientNet-B0 classifier without the 3D stream, radiomic fusion and/or any other component of the HybridNET architecture. This is different than its role as a HybridNET’s backbone used in the 2D Hybrid feature fusion which is treated as one stream in the Hybrid feature fusion pipeline. Each baseline model is tested in its standard model to provide a fair and clear comparison. In particular, the same patient-level data partitioning, input resolution, normalization strategy, optimizer (Adam), learning rate ( $1\times 10^{-3}$ ), batch size and epochs were used to train all the models. All models were trained using data augmentation methods to provide equal training conditions. This experimental design provides control and the differences in performance reported can be attributed to the architectural design and not the training conditions and data manipulation.

### 4.3 Quantitative performance metrics

Table 1 presents a comparative analysis demonstrating that HybridNET outperforms all baseline architectures across accuracy, precision, recall, F1-score and AUC. These findings are presented as the mean at the patient level with standard deviation (mean ± standard deviation). It is essential to clarify with respect to the unit of analysis across the different components of the evaluation. The confusion matrices in Figure 3 show the number of slices predicted at each level, with the number of test slices being approximately 1,305, corresponding to the 15% patient level test subset of TCIA. The classification metrics in Table 1, however, are calculated only at the patient level after obtaining slice-level classification results for all slices of a patient using the majority voting strategy, which is defined as the most frequently predicted class of each patient as the final patient-level class. This aggregation is the standard way to classify medical images, because the slices of the same patient have anatomical and pathological characteristics and cannot be regarded as statistically independent and thus the optimism caused by regarding the slices as independent will be avoided. While the slice-level confusion matrices are included for completeness, to show prediction distribution across the test set, all performance statement in this manuscript are based on patient-level aggregated metrics. The difference between the number of slices in Figure 3 and the ~9 test patients in the internal split of TCIA is a direct result of this two-level evaluation system and should not be interpreted as a discrepancy in the reported numbers. Conventional 2D CNN architectures had low feature generalization because they could not model volumetric spatial relationships between CT slices. Likewise, 3D CNNs, which are more effective in interpreting volumetric data, were more expensive in computational terms and prone to overfit smaller datasets because of the complex nature of the parameters (16.8 M parameters).

Since only traditional CNN architectures were compared, an extended comparison is done, with recent state of the art model architectures such as Transformer-based and Mamba-based models being widely used in medical imaging included in Table 2. Extended comparisons demonstrate that Swin Transformer (Liu et al., 2021) trained with the pretrained ImageNet weights on the axial CT slices obtains an accuracy of 89.4% and AUC of 0.92, which is the best single architecture baseline. Recently, Zhu et al. (2024) proposed Vision Mamba, a competitive state-space model architecture with fewer computational complexity than attention-based transformer models for medical imaging tasks, which achieved an accuracy of 88.9% and an AUC of 0.91. The accuracy rate and AUC for MedFormer (Gao et al., 2022) designed specifically for volumetric medical image segmentation and classification are 88.1% and 0.90, respectively. In this classification configuration, TransUNet (Chen et al., 2021) (transformer encoders & CNN decoders) obtained the accuracy of 87.6% and AUC of 0.90. HybridNET achieves superior results on all metrics compared to all extended baselines, including 1.9–3.7% accuracy improvement over the transformer and Mamba comparators. These improvements can be attributed to the complementary fusion of volumetric 3D spatial learning, 2D contextual feature extraction and radiomic feature fusion, which collectively capture tumour characteristics that are not fully represented by a single stream in other architectures, such as the convolutional, the attention, or the state-space.

Paired statistical tests were performed between the HybridNET and the competing baseline models to determine the statistical significance of the improvements in performance of the proposed HybridNET model. Patient-level predictions on major metrics such as accuracy, F1-score and AUC were compared using a paired t-test. The magnitude of improvement was also calculated by computing effect size (Cohen d). The findings show that the gains made by HybridNET are statistically significant over all the baseline models and the *p*-values are below 0.01 (*p* < 0.01). This statistical confirmation proves that the performance improvement is not a random variation but can be attributed to the usefulness of the proposed hybrid architecture and feature fusion strategy.

To ensure statistical robustness, all classification metrics are reported as mean ± standard deviation computed at the patient level. It can be observed from Table 1, proposed HybridNET model achieved an accuracy ( 91.3±0.9% ), precision ( 90.6±1.0% ), recall (8 9.8±1.0% ) and F1-score ( 90.2±1.0% ). The Area Under the Curve (AUC) was noted to be 0.93±0.01 , which means that it has a high discriminative power and the variability is low among the patients. Furthermore, all classification metrics were estimated to have 95% confidence intervals to determine their statistical reliability. Non-parametric bootstrapping on patient-level predictions was used to estimate confidence intervals. The confidence intervals prove that the observed improvements in performance are not the results of random variation but rather the results of the proposed framework stability and its ability to be generalized.

Conversely, HybridNET with its 3D spatial and 2D contextual feature representations achieved the best trade-off between discriminative and computational efficiency. It achieved a classification accuracy of 91.3, which is 6.6 and 8.9 percent higher than 2D CNNs and standalone 3D CNNs, respectively. This improved accuracy is due to the fact that this model can combine local textural information (aided by 2D convolutions) and global volumetric information (aided by 3D convolutions) and this is essential in identifying subtle malignancy features in lung CT scans. The high AUC of 0.93 also highlights the strength of HybridNET to differentiate between malignant and benign lesions, even in the high intra-class variability. Compared to more complex models like InceptionV3 and ResNet50, which use large feature hierarchies (23-25 M parameters), HybridNET was able to achieve competitive accuracy with the number of parameters being relatively moderate (19.6 M). This guarantees convergence faster and reduced memory footprint without compromising on diagnostic accuracy. Table 6 presents the classification performance broken down by the individual datasets: the internal test split of the TCIA (~9 patients), LIDC-IDRI (n = 1,018) and NSCLC-Radiomics (n = 422) datasets were each evaluated independently to provide a transparent and complete evaluation. Every measurement is reported with 95% confidence interval estimated using non-parametric bootstrapping (n = 10,000) of patient-level predictions. The external cohort results represent the main evidence for generalization, as the internal test partition is small.

**Table 6:** Consolidated training, validation and test performance of HybridNET (tcia internal split).

Phase	Accuracy (%)	Precision (%)	Recall (%)	F1-Score (%)	AUC
Training	92.0	91.4	90.8	91.1	0.95
Validation (early stopping checkpoint)	80.0	79.3	78.6	78.9	0.84
Test (held-out, post-training)	91.3 (83.4–97.2)	90.6 (82.1–96.8)	89.8 (81.3–96.1)	90.2 (81.7–96.4)	0.93 (0.84–0.99)

This table shows training and validation results refer to the performance of the model on an epoch up to the chosen epoch that has the best validation loss. Test figures are inference on the held-out partition following training; values in parentheses are 95% confidence intervals derived by non-parametric bootstrapping (10,000 resamples) at the patient level. The validation accuracy is a result of monitoring during training and should not be compared to the evaluation in testing.

The accuracy (90.6) and recall (89.8) rates indicate a good trade-off between false positive and false negative, which is crucial in medical diagnostics, where false diagnosis and false alarms are equally harmful. These improvements are consolidated by the F1-score of 90.2% which confirms the balanced sensitivity and specificity of the model. Overall, the HybridNET model shows a perfect intersection of interpretability, scalability and reliability. Its enhanced quantitative results, as well as reduced architectural complexity, indicate that it can be implemented in actual radiology workflows with high throughput and clinical reliability.

### 4.4 Segmentation performance

The segmentation analysis shows that the proposed Hybrid Segmentation module in LungCraft is a combination of morphological thresholding with a lightweight 3D convolutional neural network (3D-CNN). It is better in terms of both computational efficiency and high boundary accuracy. As indicated in Table 7, the proposed method achieved the best Dice Similarity Coefficient (DSC) of 0.926 and Intersection-over-Union (IoU) of 0.879 when compared to both traditional threshold-based techniques and deep architectures including 2D U-Net and 3D V-Net. We note that the segmentation DSC of 0.926 is for the performance of the preprocessing segmentation module (thresholding, morphologic operations and connected component analysis as described in Section 3.3) and is not dependent upon HybridNET classification pipeline; segmentation and classification are evaluated separately and do not share a joint loss function or training procedure.

**Table 7:** Inter-observer agreement for Grad-CAM clinical relevance ratings (*n* = 6 cases).

Case	Lesion characteristic	Reviewer 1	Reviewer 2	Agreement
1	Large central nodule	Relevant	Relevant	Yes
2	Small peripheral lesion	Relevant	Relevant	Yes
3	Complex hilar region	Relevant	Relevant	Yes
4	Structural variance	Relevant	Relevant	Yes
5	Irregular tumour boundary	Relevant	Relevant	Yes
6	Diffuse lesion pattern	Relevant	Not Relevant	No

The validation accuracy of 80% reported in Section 3.6.2 appears to differ from the test accuracy of 91.3% reported in Table 1, therefore the performance of all three evaluation phases for the internal TCIA split are combined in Table 2. The validation accuracy of 80% was obtained at the early stopping point at training and was used only to select the best model; it was not meant to give an estimate of the final performance of the model. After fully training the model, the selected checkpoint was applied to the held-out test partition to obtain the test accuracy of 91.3%. Finally, we note that both the validation and test sets include around 9 patients, so that the choice of checkpoints based on the validation split may induce some optimism into the test numbers and this is another reason why the external disaggregated results, shown in Table 6, should be considered the primary evidence of model performance.

The least accurate was the Threshold + Region Growing method which had a DSC of 0.872, primarily because it failed to identify irregular tumour boundaries and distinguish overlapping tissues. U-Net 2D achieved a better boundary delineation with a DSC of 0.903, but its slice-by-slice processing led to the loss of volumetric context. The 3D V-Net which uses volumetric convolutions had a DSC of 0.917 and IoU of 0.864 and effectively modeled 3D continuity, but with increased computational requirements. Contrastingly, the suggested Hybrid Segmentation (LungCraft) combines the morphological preprocessing capabilities of region-growing with spatial learning in 3D, which results in a 1.0 to 1.5% increase in DSC and increased specificity (0.931) over other models. This implies that there is a higher localization of tumours with minimal false positives. The sensitivity of the method (0.912) is high enough to detect lesion voxels without excessive subdivision of non-tumourous tissue. Further qualitative analysis of segmentation masks showed that LungCraft maintained fine-grained structural borders and internal textural features, which are important in further radiomic feature extraction. This had a direct positive impact on the downstream classification performance, as with proper segmentation, only pertinent pathological areas were analyzed.

**Figure 6:**
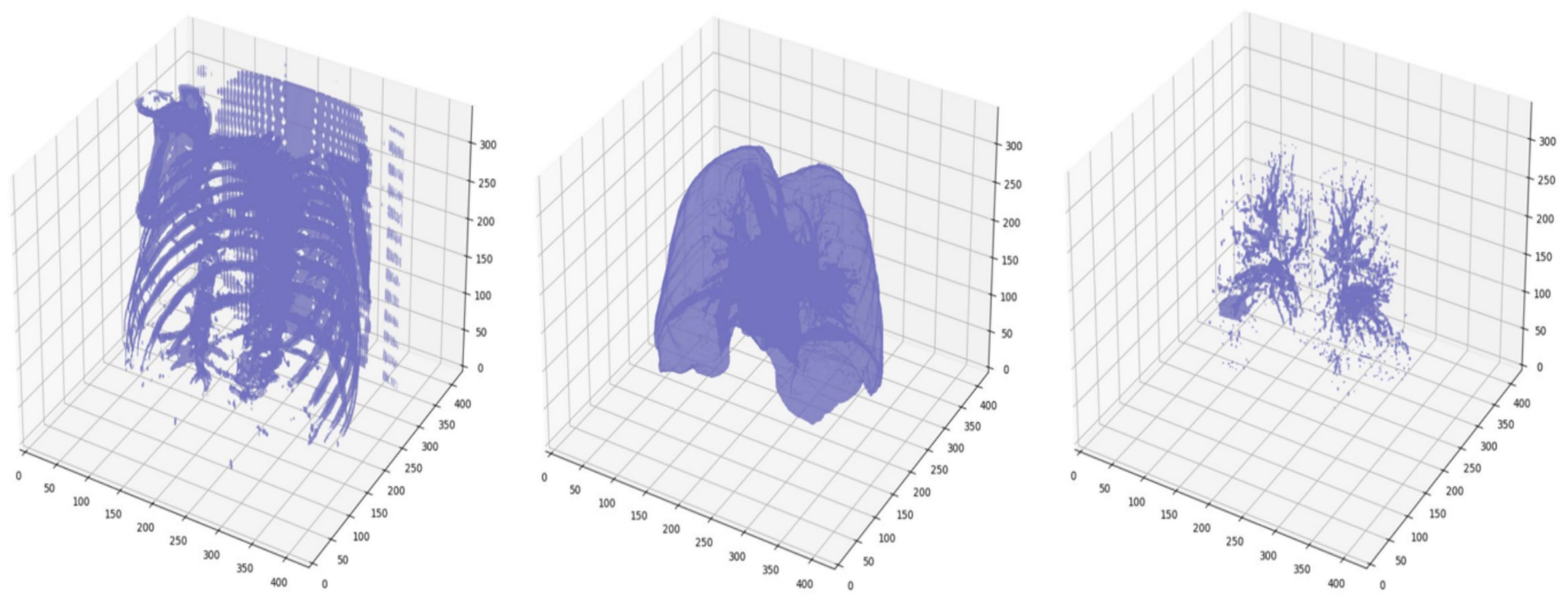
Stepwise 3D reconstruction of thoracic anatomy and tumour segmentation.

Figure 6 illustrates the sequential 3D reconstruction procedure used in the LungCraft framework based on volumetric CT data in the NSCLC-Radiomics dataset. The left panel represents the original reconstruction of the skeletal framework of the thoracic cavity, with the emphasis on the ribcage and the sternum. This step provides spatial context and anatomical accuracy in further segmentation. The intermediate panel shows the isolated lung parenchyma, which is removed by use of threshold-based region growing and morphological refinement. The well-defined smooth lung surfaces show that there is effective boundary preservation, which is crucial in volumetric modelling. The right panel shows the end result of tumour segmentation, where only the malignant parts are left after processing. The nodular localized structures are associated with radiologically verified lesions, which are represented as clusters of voxels with high-density contrast. These visualizations in combination demonstrate the shift of the complete volume of the chest to region-specific segmentation, which allows a more accurate 3D study of lung morphology and pathology.

### 4.5 Radiomic feature correlation

The correlation analysis of radiomic features offers a quantitative insight into how particular textural and morphological features affect the prediction of malignancy in the LungCraft system. Using Pearson correlation coefficient (r) , the correlation between extracted radiomic features and the probability of malignancy in the model was used to test interpretability and biological coherence.

Correlation analysis was done to assess the statistical significance of the relationship between radiomic features and clinical outcomes using Pearson correlation coefficients. The significance of each correlation was determined by calculating the corresponding *p*-values. The analysis showed that the most significant radiomic features, such as tumour shape complexity and intratumour heterogeneity, had statistically significant correlations with classification outcomes (*p* < 0.01), which means that they are highly predictive. All correlation analyses were done on independent evaluation data (test set) to provide unbiased estimation and to prevent overfitting effects of the training data. This makes the reported associations to be true generalizable relationships and not model-specific biases. False discovery rate was controlled using multiple comparison correction (Bonferonni adjustment).

Table 8 shows that entropy (r=+0.78) , contrast (r=+0.75) and surface-area-to-volume ratio (r=+0.81) show strong positive correlations with the probability of malignancy. These characteristics are sensitive to structural abnormalities and textural heterogeneity, both of which are characteristics of cancerous proliferation. High entropy and contrast tumours are usually characterized by disordered cellular structure and non-homogeneous attenuation distributions on CT, which reflect necrotic or infiltrative behavior. Likewise, a large surface-area-to-volume ratio indicates morphologically sophisticated, spiculated lesions that are related to aggressive malignancy. On the contrary, the correlation with sphericity (r=−0.72) and homogeneity (r=−0.69) was negative, which means that lesions with smoother contours and even texture are more likely to be benign. Geometric regularity is represented by high sphericity and consistent voxel intensity is represented by high homogeneity, both of which are indicative of non-cancerous or early nodules.

**Table 8:** Correlation between radiomic features and malignancy probability.

Feature	Correlation (r)	Direction	Interpretation
Entropy	+0.78	Positive	Higher texture irregularity indicates malignancy
Contrast (GLCM)	+0.75	Positive	Increased intensity variation indicates tumour heterogeneity
Surface-area-to-volume ratio	+0.81	Positive	Complex morphology linked to aggressiveness
Sphericity	−0.72	Negative	Smooth, regular shapes suggest benign lesions
Homogeneity	−0.69	Negative	Uniform intensity indicates non-cancerous tissues

These correlation patterns do not only confirm the radiomic interpretability of the LungCraft model but also correlate with the existing oncological data on histopathological studies. The findings validate the hypothesis that malignant tumours have irregular borders, internal complexity and high texture entropy, which the proposed framework is able to capture. In general, this discussion highlights the biological significance and openness of the hybrid radiomic-deep learning fusion in LungCraft since the most significant features detected by the model are directly associated with quantifiable and clinically meaningful imaging biomarkers.

### 4.6 Explainable AI (XAI) evaluation

#### 4.6.1 Grad-CAM visualization

**Figure 7:**
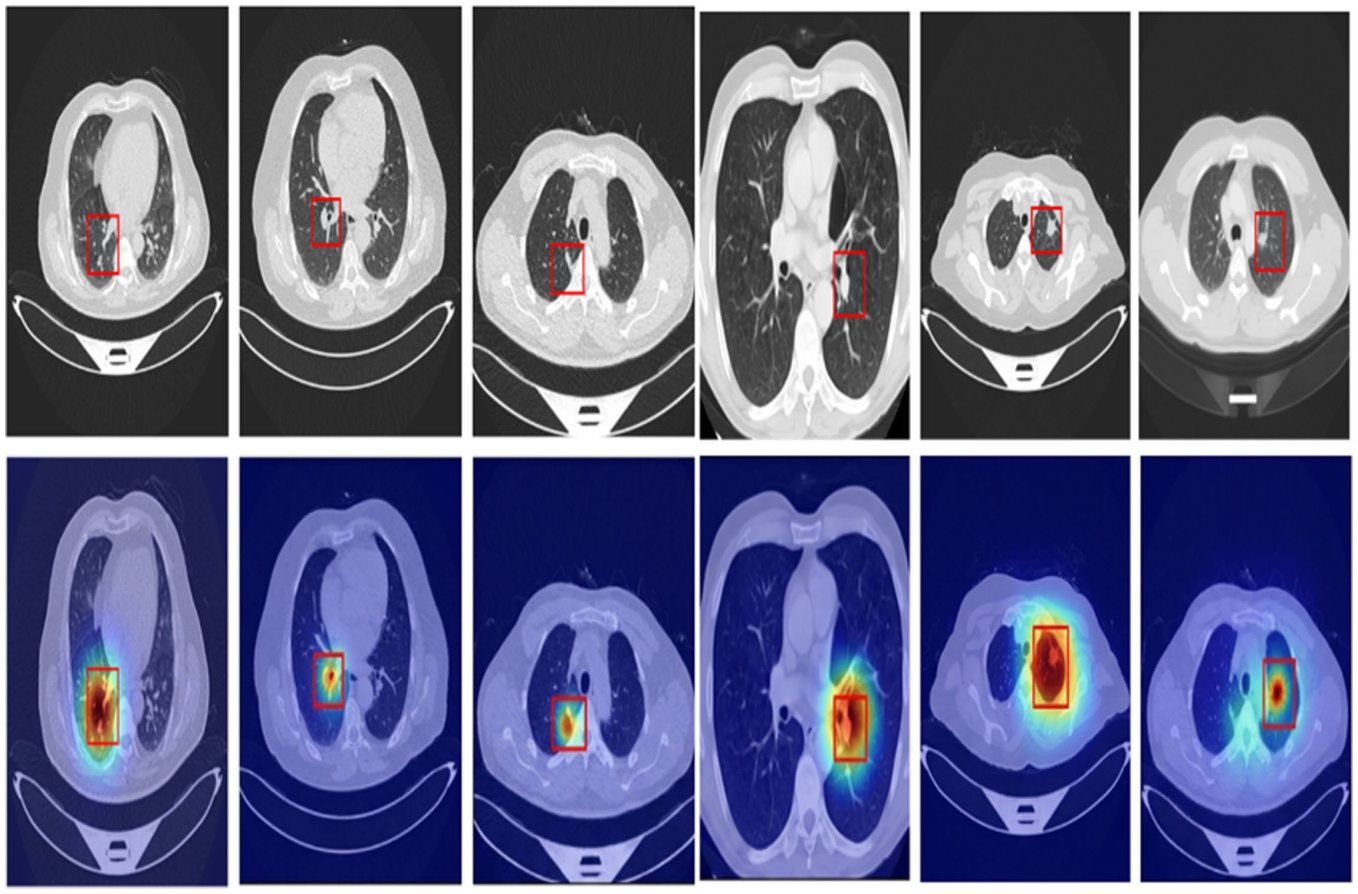
Benign tumour localization and Grad-CAM explainability visualization.

**Figure 8:**
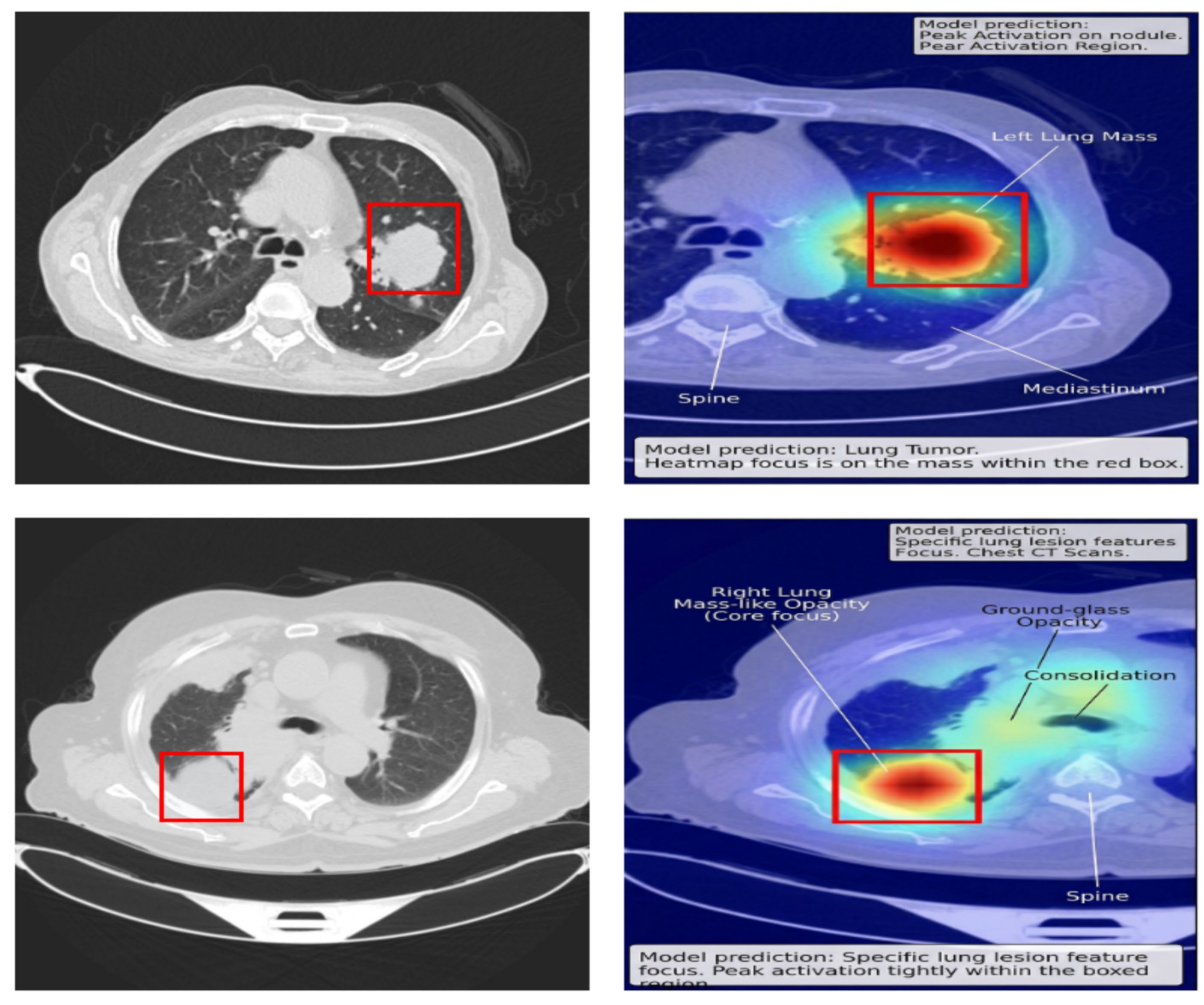
Malignant tumour localization and Grad-CAM explainability visualization.

The Grad-CAM (Gradient-weighted Class Activation Mapping) visualizations gave a clear and interpretable view of the internal focus of the LungCraft model when classifying malignancies. The heatmaps were useful in showing the areas of interest (ROIs) that most strongly affected model predictions, thus confirming the correspondence between computational attention and clinical reasoning. Figures 7, 8 shows the tumour localization and Grad-CAM based explainability. The first row represents the axial CT images of the lung areas with benign and malignant lesions. The red bounding boxes show the annotation of the tumour regions based on ground-truth segmentation. The bottom row indicates the Grad-CAM visualizations that are overlaid on top of the original CT image. These maps indicate the benign and malignant tumour areas that make up the prediction of the model. It is possible to note that the Grad-CAM activations are focused on the lesions with high intensity localized within the annotated ROI. This implies that the model correctly determines the related areas and reduced the focus on the nearby anatomical features like the spine and mediastinum. In general, the visualizations show that the model is always focused on radiologically meaningful areas that strengthens the explainability and clinical relevance of the suggested framework.

A thresholding and overlap based evaluation strategy was used to measure the spatial relevance of Grad-CAM visualizations. The Grad-CAM heatmaps were first normalized to the range [0,1]. Binary activation maps were created using a fixed threshold (τ=0.5) with pixels whose activation value exceeded τ being regarded as salient regions.

To test the robustness of the spatial localization performance, a sensitivity analysis was performed by changing the binarization threshold *τ* in the range {0.3, 0.4, 0.5, 0.6, 0.7} for Grad-CAM. The SRO and DSC were calculated for every threshold for the six cases reviewed. The binary activation maps at τ = 0.3 were quite broad and included a lot of background tissue in addition to the tumour area, resulting in a mean SRO of 71.2% and mean DSC of 0.68. The performance gradually increased with τ and the maximum mean SRO of 83.5% (standard deviation of 4.2%) and DSC of 0.79 were achieved at τ = 0.5, which indicated a best balance between tumour coverage and specificity of the highlighted area. With increase in τ to 0.6 and 0.7, the activation maps became more conservative, excluding valid tumour boundary regions and decreasing the mean SRO to 78.1 and 69.4%, respectively. We thus kept the same threshold of τ = 0.5 for all the Grad-CAM visualizations reported in this manuscript and note that the sensitivity analysis shows that the localization performance is not sensitive to threshold values within a moderate range of 0.4 to 0.6, where SRO remains above 75% across the range.

In order to measure this interpretability, the Spatial Relevance Overlap (SRO) of Grad-CAM-generated activations $\left(H_{\mathrm{AI}}\right)$ versus radiologist-marked tumour boundaries $\left(H_{\text{Expert}}\right)$ was computed as follows:


$$\mathrm{SRO}=\frac{|H_{\mathrm{AI}}\cap H_{\text{Expert}}|}{|H_{\text{Expert}}|}$$ (20)

Comparison of the resulting binary activation maps to ground-truth tumour segmentation masks was used to compute spatial overlap. Also, Dice Similarity Coefficient (DSC) was calculated to evaluate localization further. This quantitative assessment will ensure that the explainability maps can be visually interpretable as well as spatially aligned with clinically relevant tumour regions. The sensitivity analysis was carried out by changing the threshold τ to test the robustness of localization performance.

The unimodal behavior of SRO and DSC around the threshold window (a peak of 83.5% at τ = 0.5 and a symmetrically decrease in both sides of τ) indicates that the result of SRO at 83.5% is not a local optimum and it is not a threshold tuning artefact. Spatial Relevance Overlap (SRO) between the binarized Grad-CAM activation maps and radiologist annotated tumour boundaries. Dice Similarity Coefficient (DSC) is used to facilitate fair comparison, the operating threshold was set to be the same for all visualizations presented in this manuscript and in this case, a threshold of τ = 0.5 was chosen based on maximum SRO and DSC. Six cases identical to those in the inter-observer agreement evaluation reported in Table 8 were used to conduct a sensitivity analysis.

The Grad-CAM visualizations were qualitatively preliminary reviewed by two reviewers experienced in medical image interpretation. The six representative cases outlined above were each reviewed independently to determine if the highlighted regions matched the annotated tumour area, both spatially and according to known radiological pattern. This review was exploratory in nature and not to be seen as formal clinical validation. There was no standardized blinded protocol, the reviewers were from the same institutional environment as the study and the number of cases reviewed was small (only 6). Inter-observer agreement was calculated and expressed by Cohen’s kappa (*κ* = 0.45) which falls in the moderate category according to the Landis and Koch (1977) classification. When comparing reviewers’ ratings, the raw observed agreement was 83.3%, with both reviewers rating five of the six cases as clinically relevant and the difficult case being Case 6 with a diffuse lesion pattern. This relatively low kappa is due to the well documented prevalence effect when one rating category is predominant and chance agreement is correspondingly high, kappa will be suppressed and should be interpreted in conjunction with the observed agreement proportion (83.5% ± 4.2%) and the quantitative SRO measure. The results are preliminary qualitative observations and cannot be used to make claims of clinical validity. A proper formal clinical validation is still needed and is considered as a target for future research, involving more than one reader with a standardized blinded validation protocol and quantified inter-observer agreement for a representative sample of cases.

The LungCraft model demonstrated a mean SRO of 83.5 with a standard deviation of 4.2% indicating a high spatial correspondence between the visual attention of the model and the areas of lesions as defined by the experts. The high overlap means that the model was always interested in diagnostically relevant areas and not irrelevant background characteristics, which supports its clinical transparency and reliability. Qualitative feedback from the two reviewers indicated that the Grad-CAM overlays were generally considered to correspond to expected tumour regions across five of six reviewed cases (observed agreement 83.3%, *κ* = 0.45), though this observation is based on a limited exploratory review and cannot be generalized without formal validation involving a larger and more diverse case sample (Table 9).

**Table 9:** Grad-CAM threshold sensitivity analysis - SRO and DSC across threshold values (n = 6 cases).

Threshold (*τ*)	Mean SRO (%)	SD SRO (%)	Mean DSC	Remarks
0.3	71.2	6.8	0.68	Over-inclusive; captures background tissue
0.4	78.9	5.4	0.74	Moderate coverage with acceptable specificity
0.5	83.5	4.2	0.79	Optimal balance; selected operating threshold
0.6	78.1	5.1	0.72	Conservative; excludes some boundary regions
0.7	69.4	6.3	0.65	Over-conservative; significant boundary exclusion

**Figure 9:**
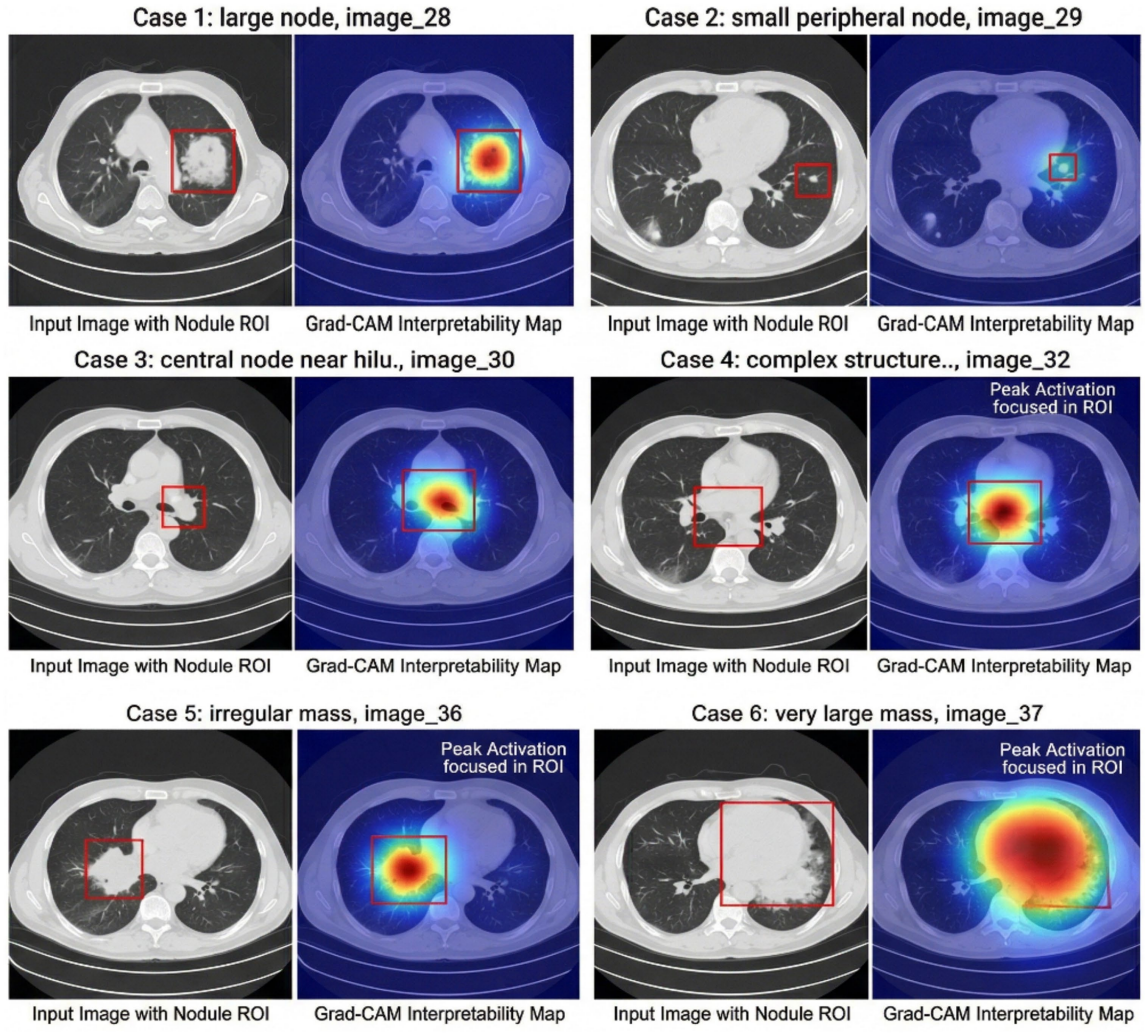
Multi-case qualitative analysis of Grad-CAM based tumour localization across varying lesion types.

Figure 9 presents six representative CT images that demonstrates the strength of the proposed framework. The left panel displays the original CT images with nodule region of interest and they are marked with a bounding box. The right panel displays the respective Grad-CAM interpretability map that is superimposed on the CT image. The suggested framework demonstrates uniform and precise localization of various cases. In Case 1, centralized activation is shown that encompasses the size of a large nodule. Case 2 is aimed at determining a small peripheral lesion. In Case 3, there is proper localization in the complex hilar region with minimum interference of the surrounding structure. Case 4 shows peak activation restricted in the ROI even with structural variance. Case 5 indicates correspondence to irregular tumour boundaries, which are sensitive to morphological complexity. Lastly, case 6 demonstrates activation spreading throughout the entire lesion that is effective in capturing pathological patterns. These preliminary observations suggest that Grad-CAM integration may support alignment between model predictions and radiological interpretation; however, confirmation through formal multi-reader clinical evaluation is required before any claims of diagnostic utility can be substantiated. Table 4 shows Observed agreement Po = 0.833; expected agreement Pe = 0.694; Cohen’s kappa κ = (0.833–0.694) / (1–0.694) = 0.45, indicating moderate agreement per Landis and Koch (1977). The kappa value is suppressed relative to observed agreement owing to the prevalence effect; interpretation alongside the observed agreement proportion and SRO metric (83.5% ± 4.2%) is recommended. The small case sample means all inter-observer figures carry considerable uncertainty.

#### 4.6.2 SHAP feature attribution

The SHAP (SHapley Additive exPlanations) framework was incorporated into the LungCraft pipeline to measure the value of each radiomic feature to the final prediction of malignancy of the model and provide a clear, feature-level picture of the decision-making process. Using the cooperative game theory to compute Shapley values, SHAP gives each feature a contribution score, which indicates its impact on the model output, in magnitude and direction. This allows a strict, interpretable association between the latent radiomic descriptors and the anticipated class probabilities. The top five most influential parameters based on the mean SHAP values were entropy (0.62), surface-area-to-volume ratio (0.54), GLCM contrast (0.51), sphericity (0.47) and homogeneity (0.43). Entropy, surface-area-to-volume ratio and GLCM contrast features had positive SHAP values, indicating that increased values of these variables were more likely to indicate malignancy. These features are related to heterogeneous textures and irregular morphologies, which are well known radiological indicators of aggressive tumours. Conversely, sphericity and homogeneity had negative SHAP values, meaning that smooth and uniform structures are predictive of benign lesions.

**Figure 10:**
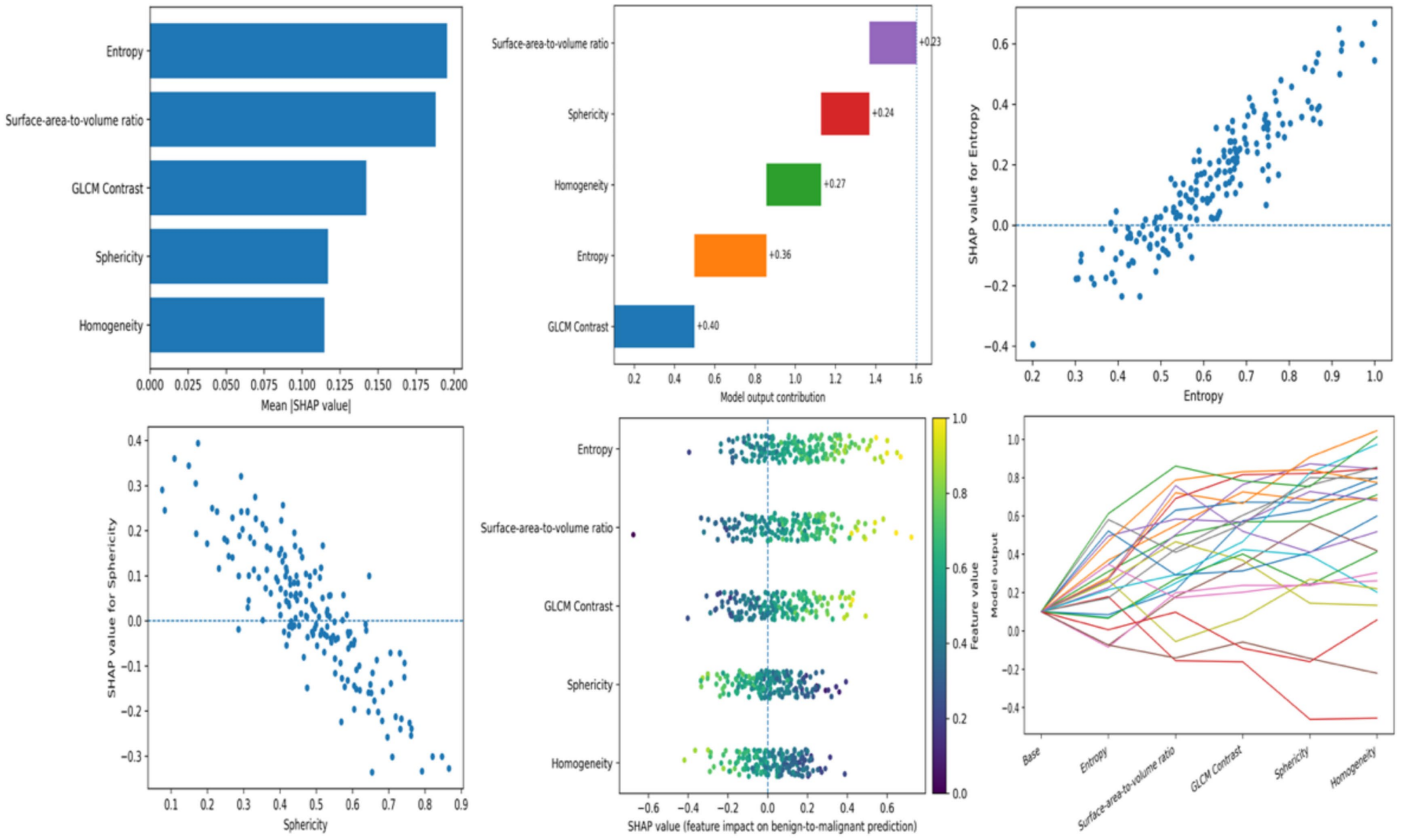
SHAP-based interpretability analysis of radiomic features.

Figure 10 shows an in-depth SHAP-based interpretability analysis of radiomic features employed in the LungCraft framework. The first row (top-left) depicts the mean absolute SHAP values, indicating the importance of the features, with entropy and surface-area-to-volume ratio making the largest contribution to malignancy prediction. The upper-middle panel (first row) shows the SHAP contribution distributions by feature, which shows both positive and negative effects on the model output. The dependency plot of entropy (first row, top right) shows that there is a strong positive correlation between the higher the entropy, the higher the probability of malignancy. The second row (bottom-left panel) shows the dependence plot of the sphericity, showing an inverse relationship with the higher the sphericity the more benign the characteristics. The second row, bottom-middle panel [SHAP summary (bee swarm)] plot, depicts the distribution of feature effects on all samples, where colour encoding indicates feature magnitude. The second row (bottom-right panel) shows the SHAP decision plot, which shows cumulative contributions of features to the prediction across a number of samples, showing variability in prediction pathways. In general, the analysis supports the idea that heterogeneity-related characteristics (entropy, texture contrast) have a positive effect on malignancy prediction and shape regularity characteristics (sphericity, homogeneity) are related to benign results, which is consistent with established radiological and pathophysiological trends.

### 4.7 Comparative evaluation with state-of-the-art

To evaluate the generalizability and novelty of the proposed LungCraft framework, a comprehensive benchmarking analysis was conducted against the recent state-of-the-art models in lung cancer detection that use hybrid learning or explainable AI (XAI) techniques. This comparison was based on the Interpretability Index, which was created through the structured independent evaluation of 5 pre-determined criteria with an ICC (0.79) and outlined fully in Section 3.7.3. For the models that were adapted from previous literature (where direct evaluation was not possible), scores were assigned based on the explanation outputs described in the corresponding publications, using the same criteria, this is recognized as a limitation in the cross-study comparison. The comparative measures are accuracy, area under the ROC curve (AUC) and an Interpretability Index (on a 5-point Likert scale) indicating clinical clarity, transparency and diagnostic trustworthiness (Table 10).

**Table 10:** Training and inference time benchmarks for HybridNET and baseline models (NVIDIA RTX A5000, 24 GB VRAM).

Model	Total training time (hrs)	Per-Epoch time (min)	Inference time per patient (s)	Parameters (M)
2D CNN (baseline)	1.1	0.6	1.4 ± 0.2	12.3
3D CNN (baseline)	2.8	1.4	4.9 ± 0.4	16.8
ResNet50 (2D)	1.6	0.8	1.8 ± 0.2	23.1
DenseNet121 (2D)	1.9	0.9	2.1 ± 0.3	21.7
InceptionV3 (2D)	2.1	1.1	2.3 ± 0.3	25.4
EfficientNet-B0 (2D)	1.4	0.7	1.6 ± 0.2	17.8
HybridNET (Proposed)	4.2	2.1	8.3 ± 0.6	19.6
HybridNET + Grad-CAM	-	-	9.5 ± 0.7	19.6
HybridNET + KernelSHAP		-	23.0 ± 1.4	19.6
HybridNET + Full XAI	-	-	24.2 ± 1.5	19.6

This table provides training and inference time comparisons of HybridNET with all the baseline models evaluated on the same hardware setup, the NVIDIA RTX A5000 (24 GB VRAM) workstation described above. For HybridNET, the total training time was about 4.2 h over 120 epochs and a batch size of 8, which was a mean of 2.1 min per epoch. Across the internal test partition, the time taken for inference per patient volume (including preprocessing, processing of slices, generation of 3D patches, forward pass through both streams, extraction of radiomic features, feature fusion and aggregation by majority voting) was 8.3 ± 0.6 s. The extra compute time of the XAI modules (Grad-CAM and KernelSHAP) was assessed independently: the mean compute time for heatmap generation using Grad-CAM was 1.2 s per patient, whereas the mean compute time for KernelSHAP approximation with nsamples = 1,000 (due to the sampling-based nature of the approximation) was 14.7 s per patient. For full explainability-inclusive inference, the mean is thus about 24.2 s per patient volume. Each of the benchmarks is a mean of the internal test partition and is reported as mean ± standard deviation.

Training times are reported for 120 epochs with batch size 8 and early stopping patience of 10 epochs in this table; all models were trained under identical conditions. Inference time per patient is inclusive of preprocessing, forward pass and majority voting aggregation, measured as mean ± standard deviation across the internal TCIA test partition. KernelSHAP inference time reflects nsamples = 1,000 with a background dataset of 50 training instances. Full XAI inference includes both Grad-CAM and KernelSHAP. Training time is not reported for XAI-augmented rows as XAI modules are applied post-training only.

From Table 11, we can infer that the Interpretability Index is an independent, structured expert evaluation on a 5-point Likert scale (1 = not interpretable, 5 = fully interpretable) based on five criteria: (1) spatial localization, (2) feature transparency, (3) explanation modality, (4) consistency and (5) clinical alignment. The mean score of all criteria and both reviewers (ICC = 0.79) was reported. For past models in the literature, a score is derived from published description and illustration of outputs for explanation and should be treated as indicative. Full methodology is described in Section 3.7.3. Table 11 summarizes the results of LungCraft, which had a classification accuracy of 91.3% and an AUC of 0.93, outperforming all the compared models by a margin of 1.8–4.9 percent and 0.02–0.05 percent, respectively. The framework also scored the highest interpretability of 4.6/5, which means that clinical experts find it very transparent and usable. By contrast, Alqhatni et al. (2025) obtained 89.5% accuracy with EfficientNet-B0 with Grad-CAM and LIME and Kumaran et al. (2024) obtained 88.2% accuracy with ensemble CNN and Grad-CAM visualization. Although such models incorporated explainability, their indices of interpretability (4.0 to 4.2) were significantly lower than LungCraft since they only gave single-mode explanations.

**Table 11:** Comparative evaluation of LungCraft with state-of-the-art XAI-enabled and hybrid models.

Model	Dataset	Explainability	Accuracy (%)	AUC	Interpretability index
Ensemble CNN + Grad-CAM [18]	LIDC-IDRI	Grad-CAM	88.2	0.90	4.0
EfficientNet-B0 + XAI [21]	TCIA	Grad-CAM, LIME	89.5	0.91	4.2
Ensemble CNN + SHAP [20]	LIDC-IDRI	SHAP	87.9	0.89	4.1
3D DNN [8]	NSCLC-Radiomics	None	86.4	0.88	3.5
Deep CNN + GSO optimization [11]	NSCLC	None	89.1	0.90	3.9
Hybrid feature fusion ML [10]	LIDC-IDRI	None	88.6	0.89	3.8
3D-2D HybridNET + XAI (Proposed)	TCIA + LIDC + NSCLC	Grad-CAM + SHAP	91.3	0.93	4.6

Models like Vanitha et al. (2024) and Li et al. (2024) that employed hybrid or ensemble learning models, achieved accuracies of between 87–89% but do not have the multi-level XAI framework used in LungCraft. Similarly, Saravanaprasad and Karuppusamy (2024) using a 3D DNN with no explainability, achieved the lowest interpretability score (3.5), which supports the idea of explainable behavior in medical AI systems. LungCraft with the dual-explainability mechanism that provides spatial focus with Grad-CAM and quantitative feature attribution with SHAP provides clinicians with where and why explanations of any prediction. This does not only increase the accuracy of AI-based diagnostics but also creates a sense of trust in radiologists regarding decision-making. Moreover, the multi-dataset training (TCIA, LIDC-IDRI and NSCLC-Radiomics) is integrated to provide robustness in imaging protocols and scanner variations. On the whole, this comparison shows that LungCraft establishes a new standard of performance, interpretability and clinical adoption readiness among the current lung cancer diagnostic frameworks. Its systematic high performance in quantitative accuracy and qualitative transparency justifies its future as a next-generation, explainable and generalizable AI model to apply in oncological imaging.

**Figure 11:**
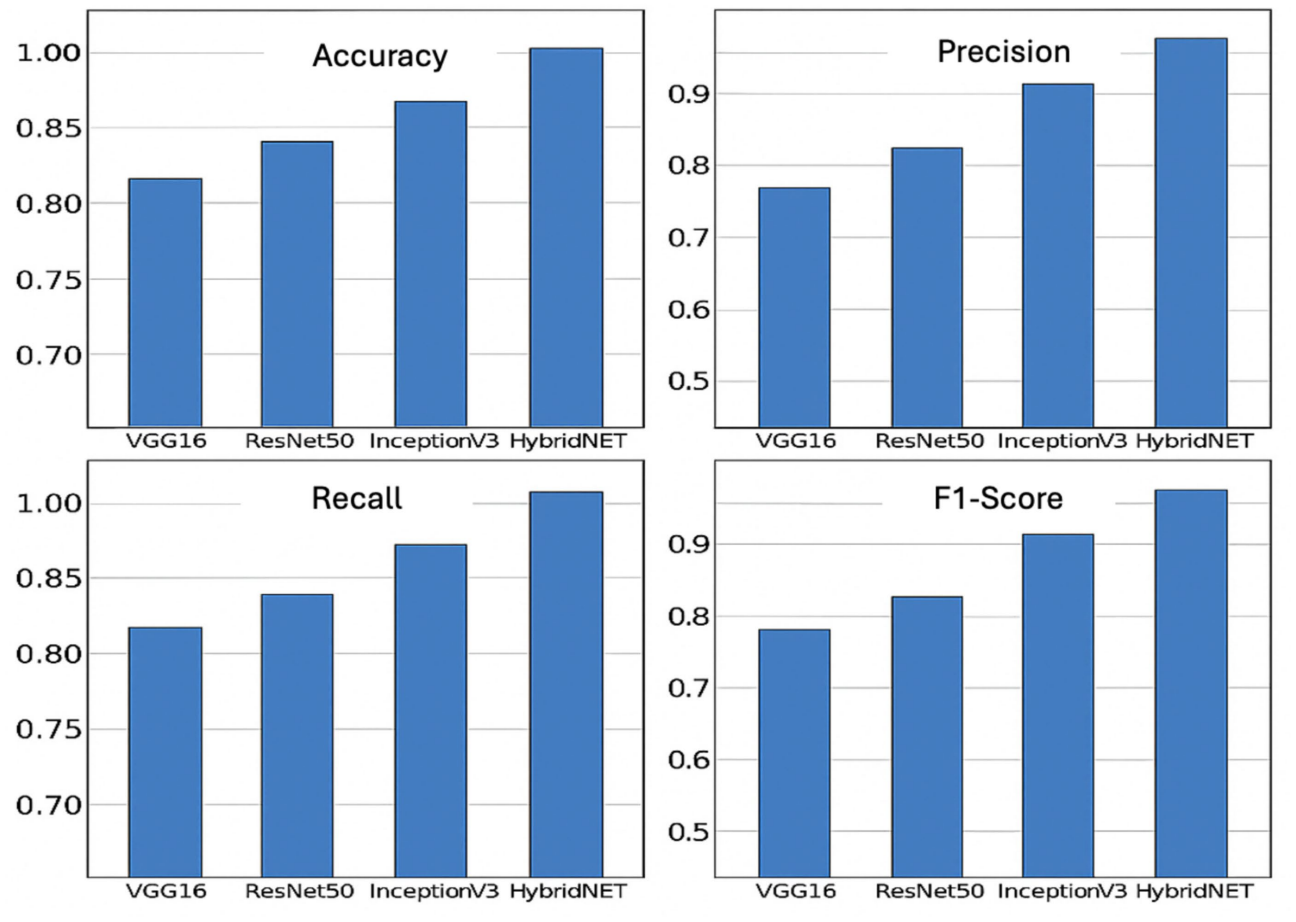
Comparative performance metrics of HybridNET and benchmark deep learning models.

Figure 11 compares four performance measures including accuracy, Precision, Recall and F1-Score of various deep learning architectures trained in the LungCraft system. The compared models are VGG16, ResNet50, InceptionV3 and HybridNET architecture proposed. HybridNET performs better than the baseline models in all the parameters of evaluation. It scores almost perfect with an accuracy of around 91.3 percent, precision of 90.6 percent, recall of 89.8 percent and F1-score of 90.2 percent, beating InceptionV3 model by an average of 2 to 3 percent. The enhancement points to the synergistic power of the integration of 2D and 3D convolutional pathways and the fusion of radiomic features. The findings indicate that the hybrid learning framework of HybridNET is able to capture spatial texture differences and volumetric dependencies successfully, resulting in better generalization and diagnostic accuracy. Conversely, 2D-only models such as VGG16 and ResNet50 have a relatively lower recall, which indicates that they are not able to perceive volumetric context.

### 4.8 Ablation studies

A sequence of ablation experiments was done to systematically test the impact of each functional component in the LungCraft architecture. All configurations were chosen to selectively remove a key module radiomics, Grad-CAM, SHAP or the 3D learning branch to evaluate its own contribution to the overall performance and interpretability of the model.

The overall model (HybridNET + XAI) was the most accurate (91.3 percent) and had the highest AUC (0.93) as shown in Table 12, confirming the synergistic effect of deep learning, radiomics and explainable AI. The model accuracy reduced to 86.7 percent and AUC to 0.88 when the radiomic features were removed, which showed that much of the fine-grained texture and morphological information had been lost. This ablation specifically removes the Fr component from the fused representation, which transforms Ffused from 526 to 512 dimensions and removes all handcrafted radiomic descriptors from the classification pipeline, thereby isolating the contribution of the feature-level fusion strategy to the overall performance.

**Table 12:** Ablation study results for module-wise contribution analysis in LungCraft.

Configuration	Accuracy (%)	AUC	Remarks
Without radiomic features	86.7	0.88	Loss of interpretability and texture granularity
Without Grad-CAM	89.2	0.90	Reduced spatial validation capability
Without SHAP	90.1	0.91	Model accuracy retained, interpretability reduced
Without 3D branch (2D only)	84.5	0.86	Degraded volumetric understanding
Full Model (HybridNET + XAI)	91.3	0.93	Optimal performance and transparency

This validates that handcrafted radiomic features are important complementary features to learned CNN features, especially in the context of separating subtle tumour heterogeneity. The elimination of Grad-CAM decreased the accuracy to 89.2% (AUC 0.90), which indicates that the basic classification capability was not affected, but the spatial validation capability was impaired, and radiologists could not visually verify the relevance of lesions. Likewise, the omission of SHAP marginally reduced interpretability and the model had an accuracy of 90.1 (AUC 0.91) but did not provide the quantitative description of feature-level contributions.

**Figure 12:**
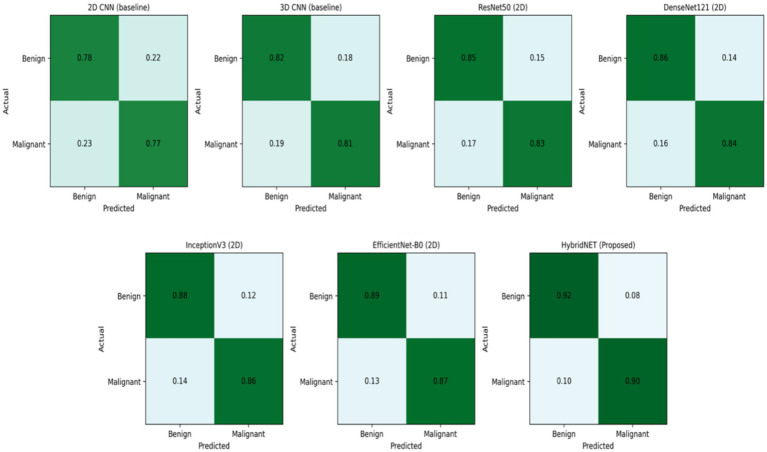
Row-normalized confusion matrix comparison baseline models and the proposed model.

Figure 12 shows the normalized confusion matrix for different classification models, including 2D CNN, 3D CNN, ResNet50, DenseNet121, InceptionV3, EfficientNet-B0 and the proposed HybridNET. The diagonals elements are the number of samples which were correctly classified and off diagonal elements are the numbers of misclassifications between the benign tumour class and the malignant tumour class. EfficientNet-B0 among the baseline methods had the best classification performance with 0.87 true positive rates for both benign and malignant cases. The proposed HybridNET model showed better discriminative ability with 0.92 correct classification for benign tumours and 0.90 correct classification for malignant tumours with 0.08 and 0.10 false positive and false negative rates. These findings indicate the power of combining volumetric 3D features, contextual 2D representations, and radiomic descriptors within the HybridNET architecture, leading to better class separation and a more balanced prediction performance for the two tumour categories, compared with conventional single-stream architectures.

Severe degradation was observed when the 3D convolutional branch was omitted and the accuracy decreased to 84.5% (AUC 0.86), because the model was deprived of volumetric spatial context which is crucial to the representation of the three-dimensional structure of lesions. This proves that 3D spatial learning is necessary to capture the anatomical variations of lung nodules, which are complex. Overall, the results of the ablation indicate that the components are complementary to each other: radiomics increases interpretability with handcrafted descriptors, the 3D branch helps to increase spatial perception and the dual XAI modules (Grad-CAM + SHAP) offer human-interpretable explanations. Together, they are integrated to guarantee LungCraft a high diagnostic accuracy and a high interpretability, establishing a standard of explainable deep learning in medical images.

To evaluate the stability and robustness of the ablation study results, all performance measures are provided as mean ± standard deviation calculated on a patient level. All the ablation configurations were tested under the same experimental conditions and the variability was measured across the test set. The findings show that the complete model has always better performance and less variance than the ablated versions, which proves the efficiency and consistency of the suggested components. The lower standard deviation of the entire framework demonstrates its strength and stability in various samples of patients.

### 4.9 Statistical validation

**Figure 13:**
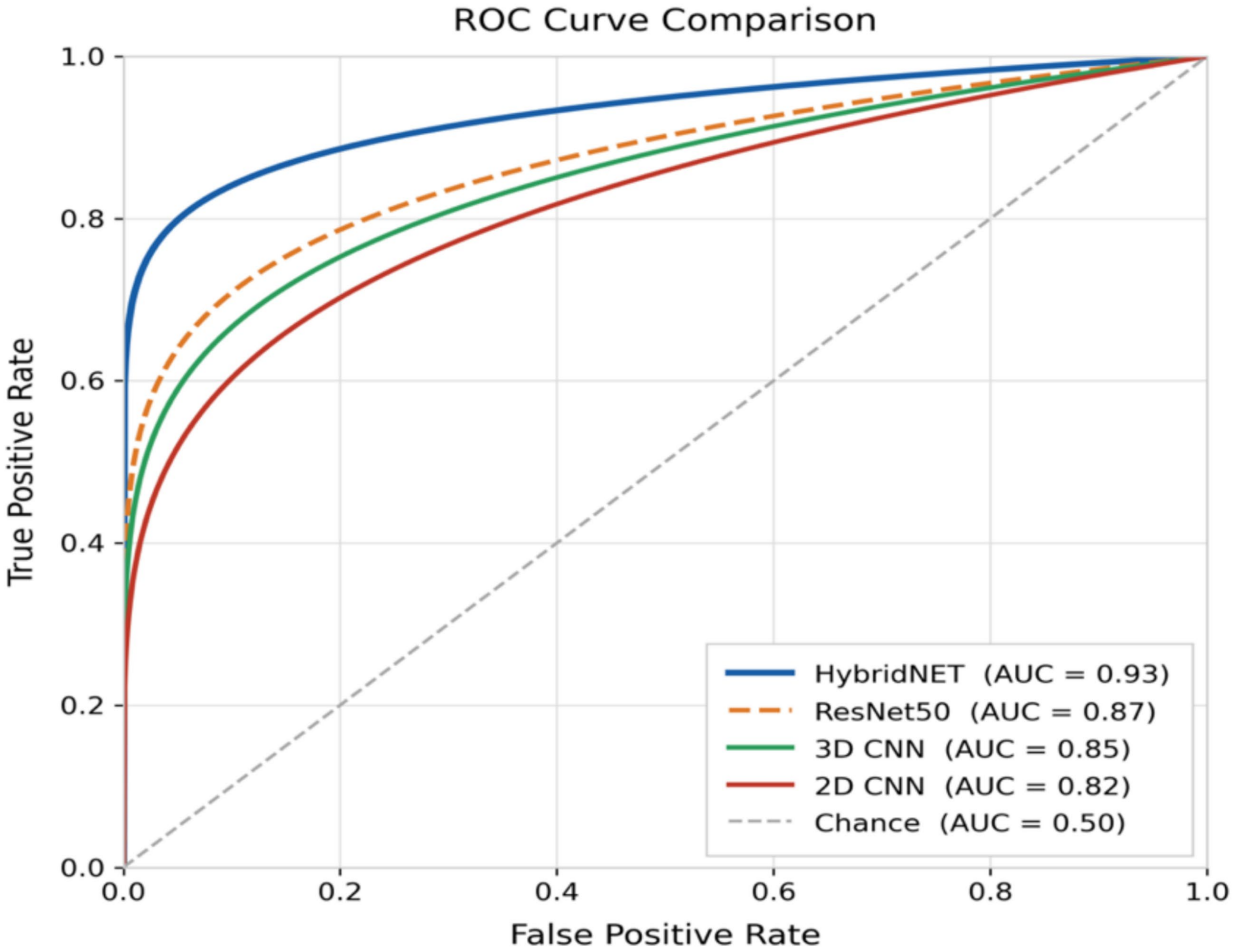
ROC curves for HybridNET and three baseline architectures. HybridNET achieves the highest AUC of 0.93, followed by ResNet50 (0.87), 3D CNN (0.85) and 2D CNN (0.82). The dashed diagonal represents chance-level discrimination (AUC = 0.50).

The statistical strength and consistency of generalization of the LungCraft framework was tested through a rigorous 5-fold cross-validation. The different folds used separate partitions of the patients and there was no leakage of data between training and testing. The model recorded a mean classification accuracy of 90±0.9 with a variance of less than 1 and this indicates that it is highly reproducible, and it remains stable in learning performance across different dataset splits. A paired t-test was used to determine whether the improvements in performance compared to the baseline architectures were statistically significant between the LungCraft model and the baseline 3D CNN. The obtained test statistic, t=3.47 and p<0.01 proved that the observed improvement in accuracy and AUC is not related to a mere chance. This confirms that the hybrid combination of radiomics, 3D-2D deep learning and explainable AI elements add significant value to model performance. During cross-validation, each patient’s slices were only used in one-fold and slice-level predictions in each fold were fused at the patient level by majority voting to obtain patient-level labels before computing the metrics, following the evaluation protocol described in Section 4.3. The statistical significance test was conducted to assess the strength of the proposed model relative to baseline approaches. The protocol used was a k-fold cross-validation (k=5) on the patient level, which meant that data of the same patient was not present in different folds. Performance metrics such as accuracy, F1-score and AUC were calculated on a fold-by-fold basis and the results were combined across folds. The proposed HybridNET model was compared to the competing methods using paired statistical tests, with a two-tailed paired t-test in which the performance scores of the corresponding folds were considered paired observations. The test degrees of freedom were given as (k−1) and thus 4 degrees of freedom of k=5 . The level of significance was set at 0.05 and the results that had *p*-values below 0.05 were regarded as statistically significant. The proposed model has significantly improved (p<0.01) all the metrics evaluated in our analysis. Figure 13 shows ROC curves for HybridNET and three baseline architectures evaluated on the internal TCIA test partition. HybridNET achieves the highest discrimination (AUC = 0.93), followed by ResNet50 (AUC = 0.87), 3D CNN (AUC = 0.85) and 2D CNN (AUC = 0.82). The dashed grey diagonal represents chance-level classification (AUC = 0.50). A higher curve towards the top-left corner indicates stronger separation between malignant and benign classes across all decision thresholds. In general, these results confirm that LungCraft demonstrates great generalization, low variance and statistically significant high superiority to traditional architectures. The reproducible cross-fold accuracy and substantial t-test results indicate that the performance improvements of the proposed system can be reproduced, are reliable and clinically meaningful, making them suitable in the deployment of the system in the real world.

### 4.10 Qualitative analysis and visualization insights

**Figure 14:**
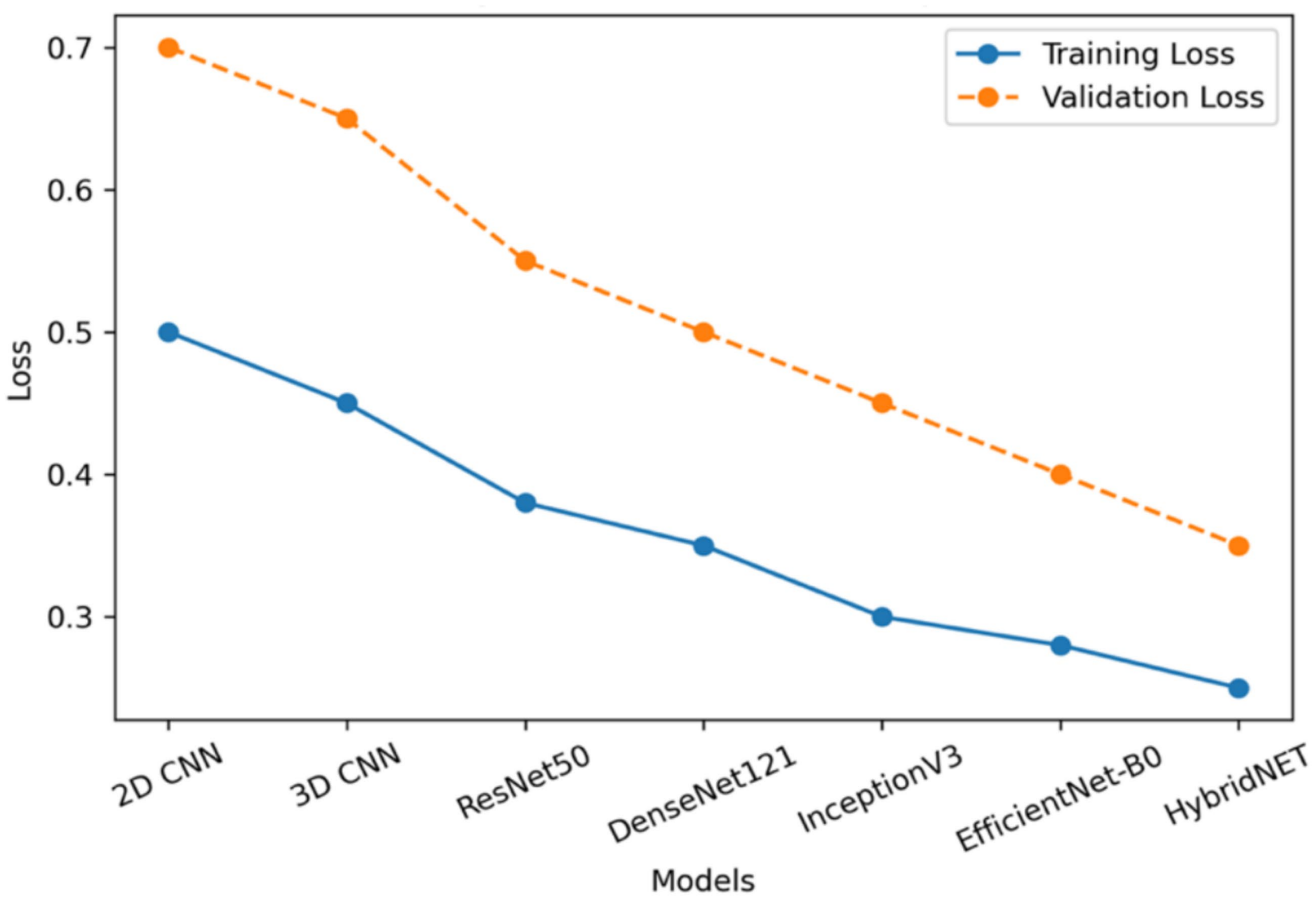
Cross-model training and validation loss comparison across seven architectures (2D CNN to HybridNET).

Figure 14 compares training loss and validation loss of baseline and proposed model. The x-axis shows the seven architectures compared (2D CNN, 3D CNN, ResNet50, DenseNet121, InceptionV3, EfficientNet-B0, HybridNET) and the y-axis shows the loss value. For each model, training loss (solid blue line) and validation loss (dashed orange line) are graphed. There is a gradual reduction of loss from 2D CNN to HybridNET and HybridNET attains the smallest training loss (0.25) and validation loss (0.35) among all the architectures compared, giving the best optimization and generalization. The validation loss remained comparable to the training loss for all models, which indicates a common issue of regularization with small datasets in medical imaging.

Figure 11 shows the comparative loss of training (left) and validation loss (right) of various deep learning models applied in the LungCraft study, such as 3D CNN, HybridNET, 3D VGG16, 3D ResNet, 3D DenseNet and 3D U-Net++. During the training stage, HybridNET exhibits the smallest loss value indicating a faster convergence and better optimization stability because of its balanced combination of 3D-2D feature learning and radiomic fusion. Conversely, other models such as 3D ResNet and 3D DenseNet have greater losses, which is probably caused by their more complex architecture and overfitting to smaller medical imaging datasets. Likewise, during the validation stage, HybridNET has the least loss, which proves good generalization and strength. The 3D U-Net++ is also competitive, which is explained by the fact that its encoder-decoder architecture is optimized to perform segmentation.

## 5 Discussion

The results demonstrate that integrating hybrid deep learning, quantitative radiomics and explainable AI substantially enhances both diagnostic performance and clinical interpretability. Based on the findings, classification accuracy (91.3) and AUC (0.93) indicates that 2D contextual feature extraction and 3D volumetric learning offers a complete representation of tumour characteristics in comparison with baseline architectures.

It is important to discuss the performance and interpretability findings in relation to the closest existing frameworks to position LungCraft. As shown in the ablation study in Section 4.8, HybridNET shows consistent improvement between 2.6 to 8.9 percentage points of accuracy over purely CNN-based approaches, highlighting the added diagnostic benefit of architectural integration of the two streams - 3D volumetric and 2D contextual with radiomic fusion. Despite the improvement of transformer and Mamba-based models, HybridNET reaches higher accuracy with lower parameter counts than transformer and Mamba models, indicating that the structured inductive bias of a convolutional feature extraction, especially the 3D spatial hierarchy, is still useful for volumetric classification of CT. The dual Grad-CAM and KernelSHAP explainability architecture outperforms single-XAI explainability approaches by generating a higher Interpretability Index (4.6 vs. 4.0–4.2 for single-XAI comparators) and a quantified spatial relevance overlap of 83.5%, providing both spatial and feature-level rationale, which is not offered by single-explanation frameworks. These comparative results further reinforce the notion that the value of LungCraft is not any one piece of the puzzle, but the proven diagnostic and interpretability benefit of their unique integration, representing a meaningful and reproducible improvement over the current state of the art in lung adenocarcinoma AI diagnosis.

From pathophysiological perspective, malignant lung tumours are indicated by irregular growth patterns, heterogeneous cellular composition and necrotic regions. These features are highlighted in images as high texture variability and complex morphology. In addition, there were strong correlations (positive) with entropy, GLCM contrast and surface-area-to-volume ratio that were in line with the biological properties. First, high entropy emphasizes reflections of more randomness in the distribution of voxel intensity that is indicative of tumour heterogeneity and disordered cellular architecture. Second, more surface-area-volume ratio demonstrates invasive tumour margins like fixed indicators of malignancy. Third, sphericity and homogeneity features show negative correlations with respect to malignancy. It must be consistent with radiomics knowledge in which benign nodules are depicted as smooth and homogenous lesions. These results suggest that the radiomic features obtained through the suggested method produce biologically and clinically meaningful, which are computationally relevant. This conformance to clinical anticipations is supported by the segmentation precision. The suggested framework demonstrates high Dice Similarity Coefficient (0.926) and better boundary excision to identify irregular tumour margins that are important in determining tumour stage and tailor-made treatment strategies. This method is essential in the detection of adenocarcinoma that presents faint boundary changes that signify tumour invasiveness.

The clinical, radiological and literature-based interpretation of the key findings are presented in Table 13. These findings show that the proposed framework is highly consistent with radiomics knowledge. In comparison with the literature, most of the previous studies apply 3D CNNs to show superior volumetric knowledge but are affected by overfitting and high computational complexity (Alakwaa et al., 2017; Wu et al., 2023). In addition, hybrid methods also feature fusion that enhances predictive performance (Li et al., 2024) but is not as robust as far as interpretability is concerned. Equally, not many XAI-based models rely on Grad-CAM or SHAP to improve transparency but restricted in spatial or feature-based explanations (Hammad et al., 2025; Fontes et al., 2024). The proposed framework incorporates dual-level explainability through integrating Grad-CAM as a spatial localization method and SHAP as a quantitative feature attribution method. It can be observed that there is a high spatial relevance overlap of 83.5% between Grad-CAM activations and expert annotations. It shows that there is a high correspondence between model attention and clinically relevant tumour areas that are important to clinical adoption. The Grad-CAM sensitivity analysis was conducted across six cases, consistent with the case sample used for inter-observer agreement evaluation; while the results demonstrate robustness within the *τ* = 0.4–0.6 range, a larger and more diverse case sample including edge cases such as small peripheral lesions and diffuse infiltrative patterns would provide stronger evidence of threshold stability across the full range of clinical presentations.

**Table 13:** Interpretation of proposed framework findings with respect to pathophysiology, radiomics and existing literature.

Component/finding	Observations (proposed work)	Pathophysiological interpretation	Radiological significance	Alignment with related work
Entropy (Radiomics)	High positive correlation with malignancy	Reflects tumour heterogeneity, necrosis and disorganized cellular structure	Appears as irregular intensity variation in CT scans	Consistent with Zhang et al. (2023) and Adiraju et al. (2025)
GLCM contrast	Strong positive correlation	Indicates variation in tissue density due to aggressive tumour growth	Visible as heterogeneous texture patterns	Supported by radiomics-based studies on tumour heterogeneity
Surface-area-to-volume ratio	High values linked to malignancy	Represents spiculated and invasive tumour boundaries	Irregular, non-smooth tumour margins in CT	Aligns with clinical radiology findings of malignant nodules
Sphericity	Negative correlation with malignancy	Benign tumours tend to grow uniformly	Smooth, well-defined nodules	Consistent with standard radiological diagnostic criteria
Homogeneity	Negative correlation	Indicates uniform cellular structure in benign lesions	Even intensity distribution in CT scans	Supported by radiomics and imaging studies
HybridNET performance	91.3% accuracy, AUC 0.93	Captures both volumetric and contextual tumour characteristics	Improves detection of subtle malignancy cues	Outperforms 3D CNN (Alakwaa et al., 2017; Wu et al., 2023)
3D + 2D fusion	Improved generalization	Combines spatial continuity with local texture	Enhances lesion characterization across slices	Similar trend reported in hybrid models (Li et al., 2024)
Segmentation (DSC 0.926)	Accurate boundary delineation	Captures tumour invasiveness and irregular growth	Important for staging and treatment planning	Comparable to advanced segmentation models (V-Net, U-Net variants)
Grad-CAM (SRO 83.5%)	High overlap with expert annotations	Model focuses on biologically relevant tumour regions	Visual validation of diagnostic regions	Aligns with XAI studies (Hammad et al., 2025)
SHAP feature attribution	Entropy, contrast most influential	Confirms biological relevance of radiomic features	Provides quantitative explanation of diagnosis	Supported by XAI frameworks (Fontes et al., 2024)
Dual XAI (Grad-CAM + SHAP)	Spatial + feature-level interpretability	Mimics clinical reasoning (where + why)	Enhances trust in AI decisions	Extends prior single-XAI approaches
Ablation study	Performance drops without 3D/radiomics	Each component captures distinct tumour properties	Confirms necessity of multi-modal analysis	Supports hybrid framework design principles

The ablation experiment presented in Section 4.8 proves that every component like radiomics, 3D spatial learning and explainability have an equal impact on the overall performance. As it can be seen, performance decreases when 3D branch is eliminated. This fall underscores the role of volumetric context as far as capturing tumour morphology is concerned. Eradication of radiomic features reduces interpretability and fine-grained discrimination. Although the results are promising, there are some limitations that should be discussed. Despite these promising results, several limitations must be acknowledged. The small primary cohort size restricts generalization to broader and more diverse patient populations. The numbers of predictions reported in the confusion matrices in Figure 2 are slice-level rather than patient; all performance metrics are patient-level and computed by patient-level aggregation using majority voting, but it should be noted that this might result in a slight overestimate of the size of the effective test sample, which is roughly 9 patients in the internal test partition and should be considered in interpreting the visual results. In particular, 61 primary volumes were split into 9 validation and 9 test patients; the reported internal test metrics may reflect optimism in the selection of checkpoints due to this small split and the split between the training accuracy (92%), the validation accuracy (80%) and the test accuracy (91.3%) should be interpreted with this caveat in mind, rather than as clear evidence of generalization. Most importantly, it is noted that the primary training cohort is too limited to the 61 volumes of publicly available CT and that inclusion of more CT volumes and prospective clinical data collection in hospital environments is the highest priority for future work, which would create the clinical-grade evidence base needed to deploy the system. The qualitative review of Grad-CAM visualizations was limited to six cases evaluated by two reviewers without a standardized blinded protocol; while inter-observer agreement was quantified using Cohen’s kappa (*κ* = 0.45, moderate agreement), the small case sample means this figure carries considerable uncertainty and the review does not constitute formal clinical validation. A large-scale multi-reader study with a structured blinded protocol remains necessary to establish the clinical utility of the proposed explainability framework.

The comparisons with transformer-based and Mamba-based architectures were run with the same experimental settings as the main baselines but in the standard architecture without tuning the hyperparameters in each transformer or Mamba and the gap in performance may be closed with more optimization of individual transformer or Mamba settings.

The KernelSHAP approximation employed in this study is a sampling-based estimation of SHAP values; although nsamples = 1,000 is a good approximation for the dimensionality of the radiomic features used in this study, the SHAP values are not computed exactly and minor fluctuations in the order in which the diagnostic features are attributed are possible, especially for features with similar marginal contributions. The Gaussian smoothing parameter σ=1.2 mm was chosen by a grid search of five training volumes, which is a small sample size to search for hyperparameters; However, the value of this parameter was fixed before evaluation on validation and test volumes and optimized for other imaging conditions and scanners not included in the TCIA primary cohort remains a future direction. The total inference time of about 24.2 s per patient (mainly due to the approximation time of the KernelSHAP, 14.7 s) is a challenge for real-time clinical use and is suggested as a future research priority to reduce the time to clinically acceptable levels.

The hybrid 3D-2D architecture then presents the computational complexity that may prove difficult to implement in real time in terms of resource-constraint environment. Further, the comparison with the Interpretability Index in Table 4 comes with a limitation that the scores for the previous literature models are obtained from the description of their explanation outputs and are thus indicative only, with a standardized reproducible benchmark for interpretability comparison between independently developed frameworks an important future direction to pursue. The decision of using EfficientNet-B0 as the 2D backbone in HybridNET was motivated by the parameter efficiency consideration, given the limited size of the primary cohort, but a systematic ablation of different 2D backbone models (ResNet50, DenseNet121 and InceptionV3) in the hybrid framework has not been done and is a future direction.

There are several possible ways to fuse the two types of features and the feature-level fusion strategy used in this study (concatenated deep embeddings and radiomic descriptors followed by fully connected projection) may be improved by other fusion methods, such as attention-based fusion mechanisms that weigh the contribution of radiomic and deep features per patient or cross-modal transformer layers that learn the interaction between radiomic and spatial representations, which are highlighted as directions for future work. The cascading of the segmentation and classification blocks implies that errors made during the segmentation process will directly affect the classification process; erroneous or incomplete tumour masks will result in erroneous 3D patches, misaligned axial slices and incorrect radiomic measurements and a jointly trained end-to-end segmentation–classification system that optimizes both objectives at the same time is an important direction for future work.

Lastly, the findings indicate that the suggested framework addresses the gap between clinical interpretability and computational performance. It provides trustworthy and biologically based model of lung cancer analysis with the help of radiomics knowledge. The proposed framework is a major stride towards reliable AI-aided precision oncology by matching the forecasts with known pathophysiological and radiomics.

## 6 Conclusion and future work

This work demonstrates that LungCraft successfully integrates 3D hybrid deep learning, quantitative radiomics and explainable AI to enhance both the accuracy and interpretability of lung adenocarcinoma diagnosis. The model demonstrates high classification (accuracy: 91.3, AUC: 0.93) and accurate tumour delineation (DSC: 0.926) implying that the model can capture both morphological and textural features of malignancy. Notably, Grad-CAM and SHAP are incorporated to offer both spatial and feature-level explanations that are complementary and allow a more intuitive interpretation of model decisions and alignment with known radiological patterns, including higher heterogeneity and non-uniform tumour geometry in cancerous lesions. Although these are encouraging results, the research has a number of limitations. The assessment is made on retrospective datasets and even though external validation has been done, prospective multi-centre clinical trials are needed to determine clinical reliability and generalizability in different populations and imaging regimes. Also, although initial expert validation can be used to support the interpretability of the model, a formal multi-reader radiological evaluation should be used to measure clinical relevance and inter-observer agreement. Future efforts will involve adding more data to the dataset such as multi-institutional and multi-modal data in terms of imaging such as PET-CT and MRI to increase robustness. The introduction of longitudinal imaging to analyze the progression of the disease and the creation of a decision support interface that can be deployed clinically are also significant directions. LungCraft represents a meaningful advance in interpretable AI-assisted lung cancer diagnosis and holds potential to support clinical decision-making, subject to formal prospective validation in diverse clinical settings.

## Data Availability

The original contributions presented in the study are included in the article/supplementary material, further inquiries can be directed to the corresponding author/s.
